# Soil microbial ecology and microbiome-metabolite linkages improve understanding of ecosystem states along terrestrial-aquatic interfaces

**DOI:** 10.1093/femsec/fiag066

**Published:** 2026-06-24

**Authors:** Sreejata Bandopadhyay, Robert E Danczak, Kaizad F Patel, Kathleen R Beilsmith, Pamela B Weisenhorn, Trisha L Spanbauer, Nicholas J Reichart, Michael N Weintraub, Vanessa L Bailey

**Affiliations:** Biological Sciences Division, Pacific Northwest National Laboratory, Richland, WA 99352, USA; Biological Sciences Division, Pacific Northwest National Laboratory, Richland, WA 99352, USA; Biological Sciences Division, Pacific Northwest National Laboratory, Richland, WA 99352, USA; Data Science and Learning Division, Argonne National Laboratory, Lemont, IL 60439, USA; Biosciences Division, Argonne National Laboratory, Lemont, IL 60439, USA; Department of Environmental Sciences, University of Toledo, Toledo, OH 43606, USA; Biological Sciences Division, Pacific Northwest National Laboratory, Richland, WA 99352, USA; Biological Sciences Division, Pacific Northwest National Laboratory, Richland, WA 99352, USA; Department of Environmental Sciences, University of Toledo, Toledo, OH 43606, USA; Biological Sciences Division, Pacific Northwest National Laboratory, Richland, WA 99352, USA; Department of Environmental Sciences, University of Toledo, Toledo, OH 43606, USA

**Keywords:** Coastal ecosystems, terrestrial-aquatic interfaces, microbiome ecology, indicator species and core microbiome, soil organic matter, microbiome-metabolite linkages

## Abstract

Coastal soils are dynamic systems where unique microbial niches are shaped by the intensity and duration of flooding between the terrestrial and aquatic boundaries of the terrestrial-aquatic interface (TAI). We aimed to understand the soil microbial community (16S rRNA gene) along the TAIs of a freshwater versus estuarine region and how it relates to organic matter (OM, via Fourier Transform Ion Cyclotron Resonance Mass Spectrometry). We studied the TAI gradients along a transect from upland (forested), transition (stressed forest), to wetland at three sites in each of the Lake Erie (freshwater) and Chesapeake Bay (estuarine) regions. Microbial communities differed significantly by region, transect position, and site. Contrary to expectations, given their dynamic hydrologies, transitions represented midpoints in microbial richness and diversity. We identified a core microbiome conserved across all transect positions within a region, highlighting potential microbial functions most resilient to environmental change. Indicator taxa unique to each transect position defined specific niches shaped by soil biogeochemistry. Co-expression networks of feature-level β-nearest-taxon indices revealed positive relationships in bacterial and OM feature contributions to community assembly. Our study provides critical insights into microbial communities at the forefront of hydrological changes in coastal areas that connect the land to lakes and oceans and remain vulnerable to changing weather patterns.

## Teaser

Soil microbial communities and organic matter composition differed between upland, transition and wetland zones of terrestrial-aquatic interfaces in a freshwater and estuarine coastal region, with positive relationships in their contributions to community assembly.

## Introduction

Coastal terrestrial-aquatic interfaces (TAIs) are critical ecosystems that are vulnerable to changing weather patterns and hydrology experiencing storm surges, hurricanes, and water level rise (Leaman et al. 2021, Cantelon and Kurylyk 2024, O’Donnell et al. 2024). Fluctuating water and salt conditions affect solute and sediment runoff and drive changes in redox conditions, with associated effects on biogeochemical cycling. Thus, TAIs represent dynamic ecosystems where the intersection of water and land can be a hotspot for a range of diverse biogeochemical functions, many of which are driven by short-term hydrologic conditions (Soininen et al. 2015, Ward et al. 2020, Regnier et al. 2022, Casas-Ruiz et al. 2023, Bowen et al. 2024).

A TAI can be categorized across a gradient of water exposure from densely vegetated upland to moderate and sparse vegetation in the transition and wetland zones (Bailey et al. 2017). The microbial community structure and function are expected to differ along this gradient due to varying levels of exposure to freshwater and saltwater, and topographic variation. The repeated inundation events and wetting–drying cycles add to the complexity of these environments and are expected to affect microbial turnover and adjustment of keystone or indicator microbial taxa. This study focuses on soil microbial communities at coastal TAIs that span the upland-transition-wetland continuum. These transect zones and the word “coastal” are not intended to be synonymous here but reflect the different parts of a coastal TAI ecosystem.

The TAIs provide a space-for-time substitution to study the impacts of hydrological fluctuations on microbial community structure, function and assembly. Furthermore, as weather and storm events initiate ecosystem state changes (ghost forest formation, wetland migration) the connections between microbial communities, biogeochemical functions, and soil organic matter (OM) can collectively address the extent of hydrological impact on these ecosystems (Chen et al. 2023). These data are also valuable for Earth System Models (ESMs) (Golaz et al. 2019, Sulman et al. 2024) that provide important predictions of future landscape changes. A detailed characterization and contrast of the microbial communities across estuarine and freshwater systems encompassing several sites within each region, provides a strong foundational dataset to improve model predictions.

Microbial communities in TAIs govern critical functions including but not limited to redox processes, greenhouse gas emissions, and elemental cycling. The key redox-affected microbial metabolisms in TAIs include aerobic and anaerobic respiration (sometimes occurring simultaneously) (Marchant et al. 2017), fermentation, denitrification, manganese and iron oxidation and reduction, sulfate reduction, methanogenesis, and methanotrophy (Chowdhury and Dick 2013, Peralta et al. 2014, Sulman et al. 2024). Decomposition rates of macromolecular substrates often have potential links to oxygen availability, which is an important consideration in oxic and anoxic environments of TAIs. While the degradation of certain compounds like lignin may require oxidative enzymes and oxygen or peroxide, the more abundant constituents of soil OM such as cellulose and hemicellulose, can be decomposed by enzymes that do not directly require oxygen (Peng et al. 2018, He et al. 2025).

Microbial communities along the TAI are likely differentially sensitive to freshwater and saltwater inundation, which makes the comparisons across these coastal regions important to consider. However, studies are mixed in this regard, with some noting differences between freshwater and saltwater regions (Li et al. 2019) and others only focusing on saltwater systems (Brown et al. 2022). Studies that utilize standardized sampling across multiple freshwater and saltwater sites are rare but necessary for finding generic and contrasting trends in microbial communities.

Microbial indicator species are sensitive to environmental perturbations and are often used as indicators of the ecological status of an environment (Ma et al. 2022). Studies in this regard often focus on a few microbial functional groups, for example, the ratio of ammonia-oxidizing archaeal (AOA) and bacterial populations has been proposed as an indicator of wetland nutrient status (Zhou et al. 2020), but they omit the context of other metabolic processes dominant in wetlands such as sulfur and iron reduction. Different indicator microbial groups can also be used for wetland assessment and management (Urakawa and Bernhard 2017). Defining microbial indicators along the TAIs of freshwater and estuarine coastal systems can identify taxa that are most sensitive as ecosystem states change from uplands to wetlands, while any overlapping species across TAI zones can be indicative of an ongoing system change. Studies that have used standardized sampling techniques across coastal TAIs leveraging space-for-time substitution are rare. While methods that evaluate the prevalence of specific microbial indicator groups such as AOA (Zhou et al. 2020) are common, the community-level context is often ignored. Additionally, core microbial communities that persist across ecosystem states are often overlooked in coastal microbiome studies but are important to understand which species will survive despite environmental change. This is critical to assess microbial functions that will be robust to rising water levels and inundation when upland and transition zones may progress towards wetlands.

The term “microbial indicators” refers to those taxa that are statistically determined to be strongly associated with a particular environment, treatment group, or habitat type (Hermans et al. 2017, Urakawa and Bernhard 2017, Ma et al. 2022). Thus, indicator taxa are considered microbial “fingerprints” of a specific condition. In this study, these environments are transect positions at the coastal TAI (upland, transition, or wetlands). We define indicator taxa (specifically OTUs resolved at the genus level) as bacterial groups that are consistently found in the upland, transition, or wetland zones. This term is borrowed from traditional plant and animal ecology, where an indicator species is defined as an organism whose presence, absence or abundance reflects a specific environmental condition (Dufrêne and Legendre 1997).

To link ecology with function, high-throughput mass spectrometry data is often used in combination with microbial sequence data, however, integration of these datasets remains a challenge. An abundance-based approach is commonly used to quantify how specific microbial taxa and molecular features co-vary in abundance, diversity, or similarity (Tanentzap et al. 2019). However, studies that prioritize linkages between the ecology of microbes and functional properties of organic molecules in a coastal ecosystem context are limited. Such ecology-function linkages are more likely than abundance-based approaches to decipher causal connections between microbial taxa and organic molecular features (Danczak et al. 2022). Previous studies have shown that ecological concepts and tools can be used to analyze metabolomes, providing insights into the factors influencing the spatiotemporal dynamics of meta-metabolomes (Danczak et al. 2020).

Our goal was to understand the common microbial features (a “core microbiome”) across the different zones of the TAI, as well as differentiating features (indicator taxa) that highlight the key differences between these zones. We characterized the soil microbial communities along TAI transects in two regions, the saltwater-influenced Chesapeake Bay and freshwater-influenced Lake Erie basin. We also assessed the soil OM profiles using ultra-high resolution mass spectrometry (UHRMS) to better understand the collective contribution of microbial and OM features to community assembly. We hypothesized that: (H1) differences in microbial community composition between TAI transects would be driven by soil abiotic factors and transect-specific bacterial indicator OTUs. However, certain taxa would remain conserved across transects denoting a core microbiome; (H2) due to the central location of the transition zone along the TAI gradient, with exposure to frequent water level fluctuations, there would be higher bacterial richness and diversity in the transition zone compared to upland and wetland zones; (H3) certain bacterial OTUs and OM features would have positive relationships in their contributions to homogenous/variable selection of their respective communities within a transect position with implications for OM turnover.

## Materials and methods

### Field sites and soil sampling

Soil sampling was conducted in the coastal regions of the Chesapeake Bay (henceforth, Chesapeake) and Lake Erie (henceforth, Erie). Three sites were sampled in each region. Along the southern shores of Lake Erie’s Western and Central basins, we sampled Crane Creek (CRC), Portage River (PTR), and Old Woman Creek (OWC), and in Chesapeake Bay, we sampled Moneystump Marsh (MSM), Goodwin Island (GWI), and the Global Change Research Wetland (GCW) (Fig. S1). Soils were collected along the coastal transects, which were denoted as wetland (closest to the waterline/shore), upland (which were further away from the shoreline with trees present), and transition (which is the middle zone connecting the upland with the wetland zones). Basic site characteristics are provided in Table S1 including latitude and longitude for sites, elevation, salinity of surface water for Chesapeake sites, mean annual temperature, and mean annual precipitation.

Soils were sampled from the Erie sites in December 2021 and from MSM and GWI in May 2022 and from GCW in September 2022. While the timing of soil collection can influence microbial communities, our timeline was driven by logistical constraints, including permitting and access to the sites. Seasonality is more important for cross-region comparisons, so this study focused mainly on transect-level trends within each region, with all Erie soils sampled at the same time. Samples were collected across the three transect locations for each site. At each transect location, soil samples were collected from 8–9 spatially distributed, discrete spots over an area of 200–1000 m^2^, and not composited to account for spatial variability. Samples were collected from the surface (O or A horizon, top 5–10 cm) and the subsurface B horizon (15–30 cm depth) where available. The samples were shipped to the laboratory on blueice and frozen at −20°C until processing and analysis. We report a subset of the soil biogeochemical data collected on these samples and used in subsequent microbiome analysis (Table S1C) with more details on site description, soil characteristics, and sampling provided in (Patel et al. 2025).

### DNA extraction and quantification

For the Chesapeake samples, DNA extractions were conducted using an automated liquid handler (epMotion 5075, Eppendorf North America). Zymobiomics 96 MagBead DNA Kit (Zymo Research Corporation, CA) was used per the manufacturer’s instructions. Precaution was taken when pipetting to ensure that the Magbeads were not disturbed. Three extractions were performed from the same soil lysate and then combined. This was followed with a DNA cleaning and concentrating step using the ZR-96 Genomic DNA Clean & Concentrator-5 Kit (Zymo Research Corporation, CA). For the Erie samples, the Zymobiomics 96 Magbead DNA isolation kit failed to generate appreciable DNAyield. Hence, a Zymo soil fecal DNA isolation kit (Zymo Research Corporation, CA) was used as per the manufacturer’s protocol. A total of 118 soil extractions were completed for Chesapeake and 87 from Erie. We did not replicate extractions for any samples.

DNA quantification was completed using a Qubit HS DNA assay (Thermo Fisher Scientific, Waltham, MA) for the Chesapeake samples and a Qubit dsDNA BR assay (Thermo Fisher Scientific, Waltham, MA) for the Erie samples. DNA quality was checked by measuring the 260/280 and 260/230 ratios on the Nanodrop (Thermo Fisher Scientific, Waltham, MA).

### 16S rRNA (V4) gene amplicon sequencing

For both the Erie and Chesapeake samples, amplicon sequencing was conducted targeting the V4 region of the 16S rRNA gene using the same forward (515f 5′-GTGYCAGCMGCCGCGGTAA-3′) and reverse (806r 5′- GGACTACNVGGGTWTCTAAT-3′) primers. However, there were some differences in the Polymerase Chain Reaction (PCR reaction set-up to normalize for the DNA concentration across both regions, which we have specified as follows. For the Chesapeake samples, PCR was performed using 5 μl of template DNA, 20 μl of Platinum II Master Mix (Invitrogen, Waltham, MA), 23 μl nuclease-free water, and 1 μl each of barcoded forward and reverse primers. Thermocycler conditions for the sequencing PCR reaction were as follows: Initial denaturation (initialization) at 94°C for 3 min, 35 cycles of denaturation (94 °C for 45 s), annealing (50°C for 60 s) and extension (72°C for 90 s), with a final extension at 72 °C for 10 min. For the Erie samples, amplicon sequencing was conducted similarly to the Chesapeake samples with slight changes to the PCR reaction composition. Namely, PCR for this set was performed using 2 μl DNA, 1 μl each of forward barcoded and reverse primers, 20 μl of Platinum II Master Mix, and 26 μl of nuclease-free water. PCR thermocycler conditions were the same as noted above for the Chesapeake samples. A negative PCR reagent control was included for both sets of samples.

Amplicon DNA concentration assay was performed in duplicate (Chesapeake) or triplicate (Erie) on each sample using the Quant-iT PicoGreen dsDNA assay kit (Invitrogen, Waltham, MA) following the manufacturer’s recommendations and 1 µl DNA per replicate. The assay was repeated twice or thrice for a given sample to estimate the DNA volume necessary for pooling and as such was not used for any statistics. Fluorescence values were assayed using a Synergy H1 plate reader, the values were averaged, and up to 200 ng of DNA was pooled for each sample. If a sample did not have 200 ng of DNA, 40 µl of that sample was pooled. The pooled sample was cleaned of excess primers using a Zymo Clean and Concentrator - 100 kit (Zymo Research, Irvine, CA). Sequencing was completed using the Illumina MiSeq platform; a v2 500 cycle kit was used with a 15% PhiX spike-in. Illumina sequencing was conducted following the manufacturer’s recommendations. The MiSeq was used to generate FASTQ files by demultiplexing the data as a final sequencing output. Further details on the sequencing protocol can be found in the Supplementary Information.

We note here that the V4 region of the 16S rRNA gene is not well-suited for studying archaeal communities, and the primers used here are not optimal for analyzing archaea. Thus, our results focus mainly on the bacterial communities rather than the archaeal communities. To limit any bias from using different DNA extraction kits for the two regions, we have conducted all analyses across transects and sites separately for the two regions. This makes our results comparable and unaffected by different kit protocols used for Chesapeake Bay and Lake Erie.

### 16S rRNA gene amplicon data processing and analysis

Sequence data were analyzed using QIIME2 (Bolyen et al. 2019) as described in (Bandopadhyay et al. 2024). All paired-end sequences with quality scores were compressed and denoised using the DADA2 plugin (Callahan et al. 2016). The Chesapeake and Erie datasets were separately denoised. The truncation parameters to use with the DADA2 plugin were determined using FIGARO (Sasada et al. 2020). The truncation length was set to 231F and 54R for Chesapeake and 91F and 194R for Erie, with minimum overlap set to 35 base pairs, which resulted in 94% (for Chesapeake) and 97% (for Erie) merging success. All truncation was performed from the 3′ end for consistent final read lengths. The resulting Chesapeake and Erie count tables were merged into a single QIIME2 artifact using the feature-table merge command. Similarly, the Chesapeake and Erie representative sequences were merged into a single QIIME2 artifact using the feature-table merge-seqs command. The representative sequences from the combined count tables were clustered at 99% identity de-novo, and the clustered representative sequences were classified using SILVA v138 (Quast et al. 2012) to generate the taxonomy file. 99% sequence identity was used to define OTUs (operational taxonomic units) to conservatively account for any potential amplification errors that may have occurred. We chose to use OTUs instead of Amplicon Sequence Variants (ASVs) because of several additional reasons such as signal dilution often observed with ASVs, prioritization of microbial functional groups versus strains in environmental gradients, and low probability of finding the exact same ASV across all samples in a group in highly heterogeneous and complex soil environments. Further elaboration on these points is documented in the Supplementary Information. The resulting OTU table and taxonomy files (clustered at 99% identity) were exported to R for ecological analysis. A phylogenetic tree was constructed by aligning amplicons using the MAFFT (Katoh et al. 2002) aligner for subsequent use in feature-level beta-nearest taxon index (βNTI_feat_) calculations and indicator OTU analysis as described below. Laboratory methods and analysis of FTICR-MS data were adapted from (Patel et al. 2021a,b) and are described in detail in the Supplementary Information.

### Ecological analyses

#### Soil microbiome analysis

The OTU and taxonomy tables were imported and analyzed in R version 4.2.3 (R Core Team 2023). For the bacterial dataset, first, any chloroplasts and mitochondria were removed from the data followed by contaminant removal using R package ‘decontam’ (Fig. S2A, B) (Davis et al. 2018). Decontam uses negative extraction controls as input to identify and remove any taxa that are classified as contaminants using a prevalence method. Reads were rarefied to an even depth of 20 000 reads across all samples. Similar details on the archaeal community rarefaction can be found in the Supplementary Information.

To better understand microbial community changes across regions, sites, and transects within Chesapeake and Erie we conducted a principal coordinate analysis based on Bray–Curtis distances to assess beta diversity. The DESeq2 package (Love et al. 2014) in R was used to denote differentially enriched taxa across the Chesapeake and Erie regions. Furthermore, we wanted to find microbial indicator species or biomarkers that defined each transect position in each region combining data across sites. For this, we used two packages to maximize our capabilities to identify such biomarkers—namely the indicator species analysis package in R (indicspecies, function multipatt, using the abundance-based counterpart of the phi coefficient called the point biserial correlation coefficient) (Chytrý et al. 2002, Cáceres and Legendre 2009), and the LDA effect size (LEfSe) package (Segata et al. 2011). Both are similar in the context of biomarker identification with indicator species being more sensitive to rare taxa compared to LEfSe which is weighed preferentially to the abundances of more common taxa. Furthermore, LEfSe identifies unique biomarkers per group only, whereas indicator species can tell us about overlapping indicators as well as unique indicators per group. We used LEfSe to identify biomarkers per transect position at the genus level, and indicator species analysis to identify indicators per transect position at OTU and genus level. We used the estimate_richness function in the R phyloseq package (McMurdie and Holmes 2013) to calculate alpha diversity metrics, namely the observed number of OTUs and the Inverse Simpson index, to understand community richness and evenness (diversity).

We also identified a core microbiome shared across upland, transition, and wetland zones across all sites within a region. To do this, we used an approach outlined in (Dueholm et al. 2022) and define core taxa as those that are present at greater than 0.1% abundance in >20% (loose core), >50% (general core), and >80% (strict core) of all samples within a region. For some overall context, we show abundance-occupancy relationships across all samples within a region using relative abundance and presence/absence information at the class level. Further details regarding indicator species and core microbiome analysis can be found in the Supplementary material.

We used soil chemistry data to understand its influence on microbiome community structure. All methods of soil chemical characterization can be found in (Patel et al. 2025). We used two separate constrained analyses of principal coordinates (CAP) analyses in package phyloseq to parse out the most important soil biogeochemical variables and FTICR compound classes that explained the variation in the bacterial community structure. Significant variables included in the final models were selected using the ordistep function from the vegan package (Barnett and Shade 2024, Oksanen et al. 2024) with the initial full model incorporating pH, specific conductance, total carbon %, total nitrogen %, total sulfur %, ammonium, nitrate, sodium, sulfate, phosphate, ammonia concentrations, and cation exchange capacity for the biogeochemical variables and aliphatic, aromatic, condensed aromatic, and unsaturated/lignin compound classes for the FTICR data.

#### Calculating feature-level β nearest taxon index (βNTI_feat_) to discern bacterial and OM features contributing to community assembly

To find metabolomic and bacterial features that show coordinated contributions to community convergence or divergence, we calculated feature-level beta-nearest taxon index (βNTI_feat_) for each OTU and OM formula (Danczak et al. 2022). We conducted a weighted gene coexpression network analysis (WGCNA) using these βNTI_feat_ values to obtain modules of features that have positively correlated ecological contributions to community assembly. Given that this metric is sensitive to the size of the input data, we calculated the βNTI_feat_ value by subsetting the data to each transect position within a region. We prioritized within transect patterns over global patterns across all transects to focus on features that drive relational similarities within transects. However, instead of the 999 permutations to generate the null model, we used 100 permutations which previously showed similar results (Quiroga et al. 2024). βNTI_feat_ was adapted from the traditional βNTI to understand the contribution of individual features to community convergence or divergence. A “feature” here is defined as any member of a community that is relevant to the study system (in this case a bacterial OTU or environmental metabolite). The values of βNTI_feat_ indicate whether a feature has an insignificant contribution to ecological variation across the metacommunity (|βNTI_feat_| < 1), somewhat contributes to ecological variation (1 < |βNTI_feat_| < 2), or significantly contributes to ecological variation (|βNTI_feat_| > 2) (Danczak et al. 2022). When βNTI_feat_ trends are negative (i.e. <−1), the feature is expected to contribute to convergence across the data. Specifically, these are features (or groups of related features) that significantly drive relational similarities across a given analytical scale. When βNTI_feat_ trends are positive (i.e. >1), the feature is expected to contribute to divergence. These include features (or groups of related features) that drive relational differences across an analytical scale (Danczak et al. 2022).

### Statistical analyses

Differences across regions, sites, and transects were visualized using a principal coordinate analysis for the microbiome data and a principal components analysis for the FTICR data. Statistically significant differences were evaluated using a permutational analysis of variance (PERMANOVA) testing the effects of main factors as well as their interaction effects. PERMANOVA was calculated in R using the adonis function in the vegan package (Oksanen et al. 2024). For the bacterial community beta diversity, the PERMANOVA model tested the effect of region, transect, and site as fixed effects. We used two different DNA extraction kits for each region, and thus the “kit” variable was perfectly confounded with region and was therefore excluded from the model formula to avoid collinearity in accordance with standard ANOVA principles and multivariate statistical analysis (Anderson 2001, Legendre et al. 2012). We tested the model with “site” included as a random effect nested within a region by using the “strata” argument to restrict the permutations within each unique site, thereby accounting for spatial autocorrelation among samples from the same site (Anderson 2008, 2017). The PERMANOVA results were similar whether we used “site” as random or fixed effect. Since we wanted to test the effect of site on the microbial community given that one of our regions have a salinity gradient across the sites, we chose to report the results which uses “site” as a fixed effect. Alpha diversity metrics were calculated separately for Chesapeake and Erie and values compared between transects using a mixed-effects model with transect position as the fixed effect and site as random effect. Post hoc analysis was conducted using a Tukey Honestly Significant Difference (HSD) test using the (Compact Letter Display) CLD function in the multcomp package (Hothorn et al. 2016) in R. Normality was checked using the Shapiro–Wilk test with a cut-off of 0.9. All data were normally distributed.

To show positive relationships between OTUs and OM features in their contributions to community convergence or divergence of the metacommunity, we used the βNTI_feat_ values to conduct a weighted gene co-expression network analysis (WGCNA) using the WGCNA package (Langfelder and Horvath 2008) in R. A signed topological overlapping matrix (TOM) was used to generate modules (networks) to preferentially select for positive correlations. Each module consists of features that demonstrate positive relationships in their contribution to community convergence/divergence. For visualization, we did not set any TOM threshold, to retain all features assigned to a module. For all network statistics, we set the TOM threshold to greater than 0.1. Further details on the network analysis are reported in the Supplementary Information.

## Results

### Lake Erie and the Chesapeake Bay have distinct microbial communities, with differences observed between transects and sites within each region

**Figure 1:**
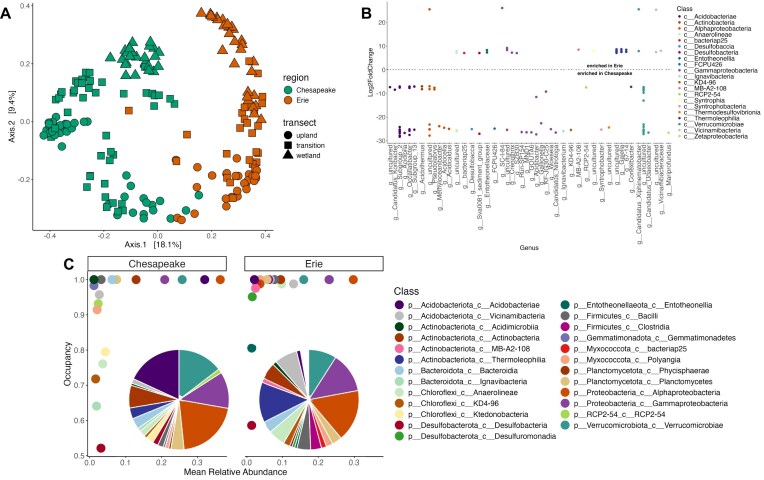
A. Principal coordinate analysis of bacterial communities showing separation by region and transect position. B. Differential expression analysis (DeSeq2) of taxa that were enriched in Erie (positive log2fold change values) or enriched in Chesapeake (negative log2fold change values). C. Abundance-occupancy distribution across all samples (combined transects and sites) for Chesapeake and Erie. We show here the twenty most abundant classes to limit possible color distinctions. Relative abundances of all classes are also visualized as a pie chart (inset).

**Figure 2:**
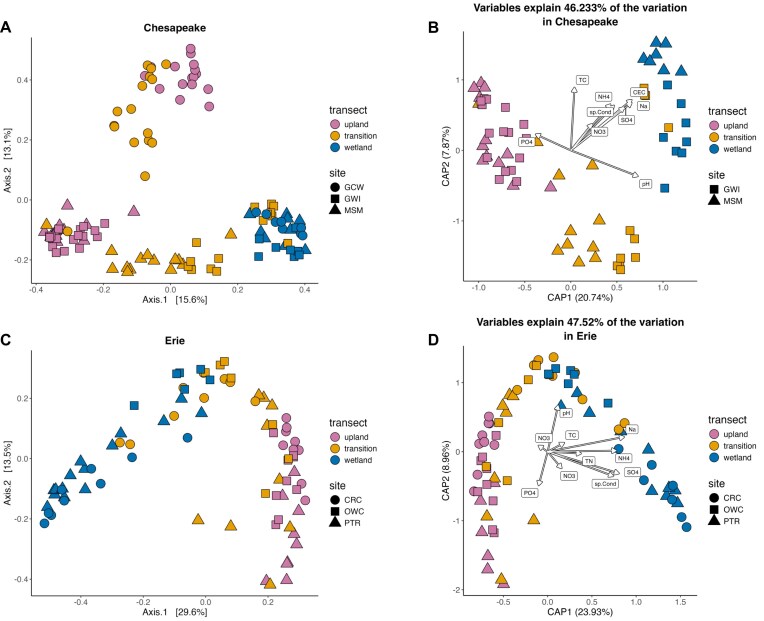
A, C: Principal coordinate analysis (Bray–Curtis distances) of bacterial community composition by transect and site in A. Chesapeake and C. Erie, and B, D: constrained analysis of principal coordinates (Bray–Curtis distances), showing the significant soil chemical variables contributing to sample distances in ordination space for B. Chesapeake and D. Erie.

The bacterial community composition differed significantly between the freshwater and estuarine coastal areas of Chesapeake and Erie (PERMANOVA *P*=0.001, *F*=60.87, Fig. 1A, full PERMANOVA results in Table 4, Supplementary Data) with a significant interaction of region with transects (PERMANOVA *P*=0.001, *F*=14.68). Additionally, samples from freshwater Erie exhibit lower dispersion compared to those from marine Chesapeake (Fig 2A). However, it is critical to note that the “region” factor was confounded with the DNA extraction Kit. Consequently, the observed differences cannot be statistically separated from potential technical artifacts introduced by the different kits. Log2fold enrichments of taxa at the OTU level (filtered to log2fold change ≥7 and ≤−7) revealed that several OTUs belonging to classes Gammaproteobacteria, Thermoleophilia, Verrucomicrobiae, and Vicinamibacteria were enriched in Erie compared to Chesapeake (Fig. 1B). On the other hand, OTUs belonging to classes Acidobacteriae, Actinobacteria, Alphaproteobacteria, Gammaproteobacteria, and Verrucomicrobiae were enriched in Chesapeake compared to Erie. Overall, certain notable bacterial classes that were higher in relative abundance in Chesapeake compared to Erie included Actinobacteria, Acidobacteriae, and Verrucomicrobiae with several samples in Chesapeake showing a predominance of Desulfobacteria (Fig. 1C, Fig. S4). Bacterial classes that had higher abundance in Erie included Vicinamibacteria and Thermoleophilia. While most of these classes had an occupancy >0.9 across all samples in Chesapeake and Erie, those that had low occupancy included classes Desulfobacteria, Ktedonobacteria, KD4-96,Anaerolineae, and Ignavibacteria in Chesapeake and Desulfobacteria and Entotheonellia in Erie. A summary of the sequence data including depth of sequencing, rarefaction thresholds and sample counts are included in the Supplementary Information.

Within each region, significant differences were observed among sites (Chesapeake *P*=0.001, Pseudo-*F*=13.51; Erie *P*=0.001, Pseudo-*F*=7.34) and transect positions (Chesapeake *P*=0.001, Pseudo-*F*=16.75; Erie *P*=0.001, Pseudo-*F*=19.001) with significant interaction effects between these factors (Fig. 2A, C, Table 4 in Supplementary Data). Overall, transects explained a greater percentage of the variation between communities compared to sites (17% versus 13% for Chesapeake, 26% versus 10% for Erie). The exception was for GCW upland and transition samples in Chesapeake, which separated notably from the other sites and transects. We also characterized the archaeal communities in Chesapeake and Erie. However, due to the low overall read counts across samples, we only chose to use the bacterial community for further analysis throughout the manuscript. We provide a brief snapshot of the archaeal community characteristics in the Supplementary Information (Fig. S5).

**Figure 3:**
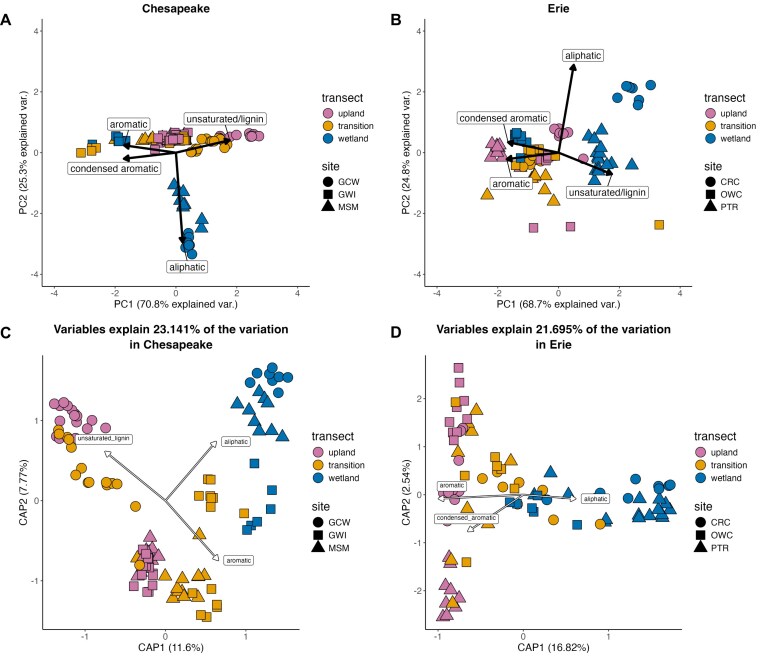
Patterns in soil OM composition across transects and sites using FTICR-MS for Chesapeake and Erie. PCA biplots depicting the organic compound classes that strongly influence the OM profiles in A. Chesapeake and B. Erie samples. Constrained ordination depicting the influence of organic compound classes on soil bacterial communities in C. Chesapeake, and D. Erie.

Using a constrained analysis of principal coordinates, we found several soil chemical characteristics that explained up to 46% and 48% of the variation in bacterial communities in Chesapeake and Erie respectively (Fig. 2B, D). We note here that GCW site was dropped in the constrained ordination for Chesapeake due to greater dispersion observed among samples when GCW was included as shown in Fig. 3A. In Chesapeake, we found cation exchange capacity, sodium, ammonium, sulfate and nitrate ion concentrations, pH, and specific conductance correlated with wetland communities. In Erie on the other hand, we found that pH and nitrate correlated with transition and wetland communities, while sodium, ammonium and sulfate ion concentrations correlated to wetland communities.

### Alpha diversity increased linearly across coastal transects

We found that both richness (number of observed OTUs) and diversity (Inverse Simpson index) were significantly higher (*P*≤0.05) in the wetland areas compared to upland and transition zones consistently across all sites in both Chesapeake (Fig. S6A, B) and Erie (Fig. S6C, D). All the transects significantly differed in their alpha diversity from each other across both regions (Tukey HSD, at *P*≤0.05) with a clear linear relationship in these responses.

### Indicator OTUs and biomarkers at the transect level

LEfSe revealed 23 transect biomarkers for Chesapeake (Fig. S7A) and 14 for Erie (Fig. S7B) at the genus level. These included several uncultured genera in upland Chesapeake, with notable wetland Chesapeake biomarkers including genera *Desulfatiglans, Methyloceanibacter, Desulfobacca* among others. Upland Erie biomarkers included genera *Candidatus Udaeobacter* (candidate genus *Udaeobacter*, family Chthoniobacteraceae)*, Mycobacterium*, and *Acidothermus* with wetland Erie biomarkers including an uncultured genus from family Methyloligellaceae, and *Sva0081_sediment_group*.

We detected several indicator OTUs that were uniquely associated with each transect position (Fig. S8, Venn diagrams), and hence, chose to plot only the 20 most abundant indicator taxa (Fig. S8, trees). Interestingly, we only found shared indicator OTUs across upland and transition, and transition and wetland zones with no overlapping indicators seen between uplands and wetlands and no indicators shared across all three transect zones. While some of the most abundant indicators in Chesapeake and Erie belonged to the same phyla (Acidobacteriota, Actinobacteriota, Proteobacteria, Verrucomicrobiota) some were unique to Chesapeake (Bacteroidota, Desulfobacterota) and Erie (Chloroflexi, Firmicutes, Myxococcota). At the OTU level, we found candidates belonging to genera *Candidatus_Udaeobacter* (upland indicator in Chesapeake and Erie), *Methylocystis* spp. (wetland indicator in Erie), *Mycobacterium* spp. (upland indicator in both Chesapeake and Erie), *Sva0081_sediment_group* spp. and *Flavobacterium* spp. (wetland indicator in Chesapeake). The total number of shared and unique OTUs per transect per site is shown in Fig. S9. The unique OTUs across transect positions differed per site with a larger number of unique OTUs detected for wetland regions in both Chesapeake and Erie. The Erie sites, however, harbored a much larger number of shared OTUs as compared to the Chesapeake sites.

We also aggregated responses at the genus level for the transect-specific indicator analysis and then compared the full set of indicators to the full LEfSe set to see if certain indicators were detected across both methods in specific transects. We found 569 transect-specific indicators at genus level that were successfully classified in Chesapeake with 432 genus-level indicators in Erie. LEfSe provided 23 transect-specific biomarkers in total for Chesapeake and 14 in Erie. Among these, upland Chesapeake had two matches across both methods (one genus *Acidibacter*, and another *AD3*, phylum Chloroflexi). There were no matches in Chesapeake transition across the two methods. However, Chesapeake wetland had several matches including genera such as *Ignavibactrium, Sva0081_sediment group, Desulfobacca, Desulfatiglans, Methyloceanibacter, Zixibacteria*, and *Spirochaeta*. In Erie upland, there were three shared genera across both indicator and LEfSe analysis namely genera *67–14* (phylum Actinobacteriota), *KD4-96* (phylum Chloroflexi), and one belonging to family *Entotheonellaceae*. No shared indicators and biomarkers were seen for Erie transition with four matches seen across both datasets in Erie wetland, namely *Bacteroidetes_vadinHA17, Sva0081_sediment_group, Syntrophorhabdus*, and *SB-5* (order Bacteroidales). The overlapping biomarkers and indicators detected across both methods provide a more streamlined pool of bacterial taxa that could be important targets for community functional analysis and explorations.

### Shared microbiome membership across transects reveal a core microbiome with potentially conserved functions across a TAI gradient irrespective of site

We found a total of 112 bacterial genera that formed a core in Chesapeake and 72 genera that belonged to a conditionally rare or abundant group (CRAT) (Fig. S10A, Fig. S11A). In Erie, there were 185 bacterial genera in the core microbiome and 13 CRAT genera (Fig. S10B, Fig. S11B). The strict core comprised only 9 bacterial genera in Chesapeake (Table S2A) compared to 29 in Erie (Table S2B). Common to both regions, we find the genus *Mycobacterium* spp. and *Pseudolabrys* spp. as a strict core member, with the others being unique to each region. While *Bacillus* spp. and *Haliangium* spp. were part of the general core in Chesapeake, they formed part of the strict core in Erie. In Erie, we also found several rhizobia species and *Geobacter* spp. forming part of the strict core.

### OM composition across regions, sites, and transects

Chesapeake and Erie also differed based on their OM composition (Fig. S12). A principal components analysis revealed that separation along PC1 was driven by aromatic and unsaturated/lignin-like compounds while separation along PC2 was largely influenced by aliphatic compounds (Fig. S12). Within Erie, wetland samples showed a stronger influence of unsaturated/lignin-like and aromatic compounds as opposed to Chesapeake where wetland samples were largely influenced by aliphatic compounds (Fig. 3A, B). Combining data across regions, we found that site explained the greatest variation in the OM profiles (PERMANOVA *R*^2^=27.5%, *F*=47.3, *P*=0.001), followed by region (PERMANOVA, *R*^2^= 6%, *F*=41.3, *P*=0.001) and transect (PERMANOVA *R*^2^ =4%, *F*=14, *P*=0.001). Transects differed largely based on region (PERMANOVA region/transect interaction *R*^2^= 0.11, *F*=37.4, *P*=0.001) but less so based on site (PERMANOVA transect/site interaction *R*^2^=0.1, *F*=8.8, P=0.001). In certain sites, we observed a strong influence of transects on FTICR feature distribution along the Van Krevelen plot (unique+shared features across transects, Fig. S13; unique features across transects, Fig. S14).

Using a constrained ordination of principal coordinates, we found that aliphatic, aromatic, and unsaturated/lignin compounds strongly influenced the bacterial communities of GCW, GWI, and MSM wetlands, MSM and GWI upland and transition, and GCW upland and transition zones respectively in Chesapeake (Fig. 3C, D). In Erie, aromatic and condensed aromatic compounds strongly influenced the bacterial communities of upland and transition zones while aliphatic compounds strongly influenced the wetland communities (Fig. 3C, D). Overall, we found these classes explained around 23% and 22% of the bacterial community variation in Chesapeake and Erie, respectively.

### Correlation networks reveal bacterial OTUs and organic molecular features that contribute to ecological processes in a coordinated way

Seventy-one modules were obtained in Chesapeake upland, 30 modules in Chesapeake wetland, 79 modules in Erie upland, 59 modules in Erie transition and 5 modules in Erie wetland. We focus here only on those modules which detect OTUs either alone or together with OM features. We found modules with both OTUs and OM features in all transects across both locations except Chesapeake transition and Erie wetlands (3 modules in Chesapeake upland, 11 modules in Chesapeake wetland, 25 modules in Erie upland, 3 modules in Erie transition). While the Chesapeake transition did not yield any modules, the 5 modules detected in Erie wetlands consisted of only OTUs without OM features. Overall, around 81% of all modules contained only OM features, with 2% modules containing only OTUs, and 17% containing OTUs and OM features.

**Figure 4:**
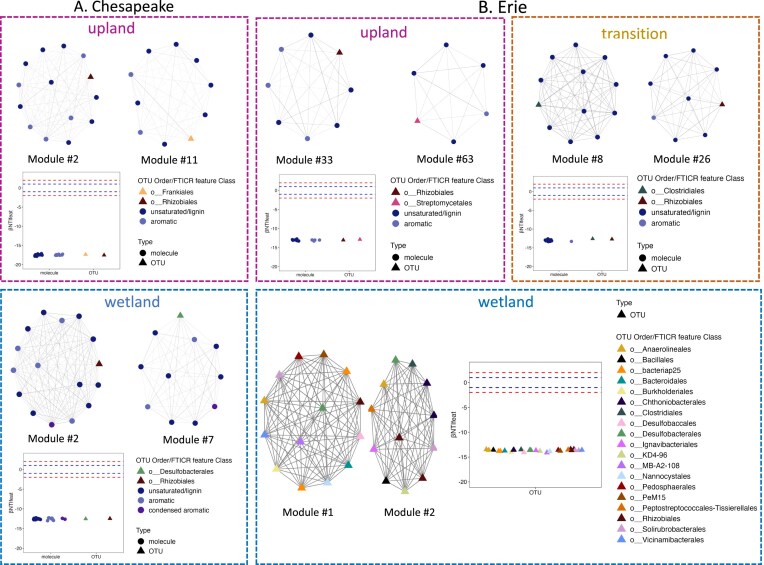
Networks (modules) generated from feature-level β-nearest taxon index (βNTI_feat_)-based WGCNA. Node colors indicate the different orders of bacteria or molecular formulae identified in modules created using WGCNA, and node shapes differentiate bacterial OTUs and molecular formulae. Modules shown in the figure are based on a signed topology overlap matrix (TOM) calculated from an adjacency matrix using the WGCNA package. TOM thresholding was not conducted for visualization to retain all features within modules. All module statistics are in Table 6, Supplementary Data, and were calculated at a TOM threshold of 0.1. These include correlation (*R*^2^) of individual features to module eigenvalues, estimates of closeness, betweenness, degree and hub score. Network edge size (width of the connecting lines between nodes) was weighted using the TOM similarity index. We plot here only a subset of modules per transect where features showed strong correlation to module eigenvalues. The graphical representation for transect-specific modules shows the specific βNTI_feat_ values for the OTUs and organic molecular formulae. The βNTI_feat_ values of all individual features across all transect-specific modules in both regions is provided in Supplementary Fig. S12 where we have cross-referenced the individual features shown in this subset of modules to show their contribution to community convergence or divergence. A |βNTI_feat_| <−2 indicates that the feature contributes significantly to community convergence, and a |βNTI_feat_| > 2 indicates that the feature contributes significantly to community divergence. The blue dashed line in the graph at +1 and −1 represents the “Contributes” threshold, while the red dashed line at +2 and −2 represents the “Significantly Contributes” threshold.

For each transect position, we focus on modules which showed a strong correlation of features to module eigenvalue and consisted of OTUs and OM features (Fig. 4). We found that nitrogen fixing bacteria such as those belonging to orders Frankiales and Rhizobiales, and unsaturated/lignin-like and aromatic compounds showed coordinated contributions to community convergence/divergence in Chesapeake upland. While Rhizobiales, unsaturated/lignin-like, aromatic and condensed aromatic classes were part of modules in Chesapeake wetland, other orders such as Pirellulales, Solirubrobacterales, Ignavibacteriales, Desulfobacterales, Anaerolineles were also predominant (Table 6, Supplementary Data). In Erie, upland modules included orders Cthoniobacterales, Streptomycetales, Corynebacteriales, Rhizobiales, and aromatic, unsaturated/lignin-like classes (Fig. 4, Table 6, Supplementary Data). In Erie transition modules, Micrococcales and Clostridiales showed positive relationships with unsaturated/lignin-like and aromatic compounds in their contributions to community assembly. Interestingly, the wetlands in Erie only contained modules with OTUs and no OM features but all OTUs were highly correlated as per the TOM similarity index (edge size weighted by TOM index in Fig. 4, Table 6, Supplementary Data). We found that members of the order Desulfobaccales and Desulfobacterales (important S-cycling members) showed coordinated ecological contributions with members of orders Anaerolineales, Solirubrobacterales, and Rhizobiales. While Burkholderiales showed correlations with all these orders, they were also associated with Clostridiales and Geobacterales (modules 3, 5). Furthermore, we found that the OTUs detected in the transect-specific modules shown in Fig. 4, were all part of the indicator OTU pool for that specific transect position, except for the OTU belonging to order Rhizobiales in Erie upland, and OTU belonging to order Clostridiales in Erie transition. Most, though not all, of the OTUs in the Erie wetland modules were also part of the indicator OTU pool. Summarizing these modules, we found that nitrogen fixers have a more ubiquitous relationship with OM features across the TAI gradient, but sulfate reducers predominantly affect microbiome assembly in the wetlands (Table S3). The transect-level βNTI_feat_ values of all OTUs and OM features are shown in Fig. S15.

## Discussion

### Distinct microbial communities by transect and site

The bacterial communities in Chesapeake and Erie soils were distinct with significant separations based on site and transects in both regions. We hypothesized that differences in microbial community composition between transects would be driven by soil abiotic factors with unique bacterial indicator OTUs associated with specific transect positions (H1). While the transect separations were dominant compared to sites, notable in Chesapeake was the separation of GCW upland and transition bacterial communities from other sites and transects. Site-specific trends were more apparent in Chesapeake than Erie, a pattern that may be explained by the fact that Chesapeake sites varied in salinity in addition to variation in hydroperiod, a feature that applies to both regions. Among Chesapeake upland sites, the GCW upland site is relatively high in elevation and has never been exposed to estuarine water storm surge to our knowledge, while the MSM and GWI upland sites are at low elevations that occasionally flood on extremely high tides. Steep topography may also explain the separation of the GCW transition zone which is more fresh (lower salinity) than the MSM and GWI transition zones. GCW samples were also all A/B horizon soil (less organic) as opposed to the other sites which included O horizon soils (organic) (Patel et al. 2025). Finally, the differences between GCW and the other sites could also be due to sampling time—GCW samples were collected in September 2022 whereas the samples from MSM and GWI were collected in May 2022. The different timing of sample collection may have exposed MSM and GWI samples to similar environmental conditions compared to GCW in turn affecting salinity of samples.

### Alpha diversity increases along the TAI

We hypothesized that alpha diversity would be highest in the transition zones (H2), reflecting hydrologic fluctuations that create a diverse microbial community with increased tolerance to wetting–drying cycles. However, contrary to our hypothesis, microbial community diversity was highest in the wetlands in both regions across all sites, indicating that the continuous exposure to estuarine water and freshwater in wetlands led to the assembly of a diverse community capable of thriving in a nutrient and moisture-rich environment. This is also corroborated by the strong correlations we observe between the soil biogeochemical analytes and the wetland soil bacterial communities in both regions. This is opposed to another study where sites closer to the open water showed decreased alpha diversity compared to sites further away from the water, though, notably, this correlated with a descending salinity gradient with highest salinity near the water likely causing a selective enrichment of microbial species in wetlands (Li et al. 2019). However, results in this regard are mixed, with another study showing that increases in soil salinity in a coastal grassland system increased alpha diversity (Brown et al. 2022). The high richness and diversity in transition and wetland zones in our study are likely linked to the relatively high contribution of several redox active compounds in influencing the transition and wetland microbial communities. The wetland transect position in this study is also a hyporheic zone (HZ) where the groundwater meets the surface water. HZs are hotspots for biogeochemical and microbial activity (Hou et al. 2017, Stach et al. 2025, Wang et al. 2025), and thus further supports the observation regarding the high alpha diversity in our wetland samples.

Transition zones have been defined previously as “hybrid ecosystems” serving as conduits for rapid fluxes of materials and energy from one adjacent well-defined ecosystem to another (Ewel et al. 2001). These zones are influenced by the ecosystems they link while possessing some unique plant and microbial species. Similarly, the coastal transition zone can represent patterns intermediate between the upland and wetland endpoints of the TAI. While the alpha diversity metrics did not support our initial hypothesis, they suggest that transition zones could be a midpoint between upland and wetland zones with measurable linear trends. This linear trend is corroborated with certain soil chemical variables, measured at these same synoptic sites, such as total S and SO_4_–S, which increased in concentration from upland to wetland zones (Patel et al. 2025). However, not all analytes demonstrated this trend, with several showing a transition hotspot. Regardless, this can have implications for coastal transition zones as a midpoint/control point in the upland to wetland continuum.

### Direct and indirect effects of abiotic and biotic factors on wetland soil microbiomes

Overall, we found that abiotic factors such as soil pH, sodium, nitrate, ammonium, phosphate and sulfate concentrations explained almost half of the variation in microbial community composition in both Chesapeake and Erie (Fig. 2B, D). Several of these analytes were strongly correlated to the wetland microbial communities. The wetland zone, being closest to the open water along the TAI, encounters frequent exposure to the estuarine and freshwater, likely contributing to the strong impact of water-influenced soil biogeochemically active solutes on wetland microbiomes. This, coupled with the presence of a HZ in our wetland transect location, explains the strong influence of wetland soil biogeochemistry on microbial community structure and diversity. Transition zones, being at the intermediate position along the TAI, are also similarly exposed to estuarine and freshwater during repeated wet-dry cycles, likely leading to a significant influence of soil solutes on a few transition-zone bacterial communities (Fig. 2B, D). Abiotic factors may have a direct or indirect effect on soil microbial communities (Lammel et al. 2018, Zhang et al. 2024), either of which influences biogeochemical functions related to C, N, P and S cycling at the TAI.

Wetland soil microbiomes can also be directly influenced via biotic factors, for example, through plant-microbe interactions, an aspect that we have not considered in this study (Prasse et al. 2015, Li et al. 2021, Song et al. 2024). It has been shown that plant-microbial feedback in response to flooding can be mediated by plant genotype (Tang et al. 2021). Given that coastal areas harbor plants with a diversity of traits that influence the dominant redox couples available to support microbial respiration, such effects of plant trait variability within or across plant species have the potential to suppress or enhance the impacts of water-level rise on microbial communities. Since our sites have distinct vegetation patterns (Table S1A, B), this is an important consideration for future work.

### Indicators of TAI ecosystem states

We identified several indicator taxa of interest in this study, as predicted by H1. For example, *Candidatus_Udaeobacter* (upland indicator in Chesapeake and Erie, Fig. S8, Table 5 in Supplementary Data) has the potential to utilize H_2_ as an alternate electron donor (Willms et al. 2020). It is generally not involved in symbiotic interactions as inferred from a lack of significant correlation between the abundance of this taxon and other prokaryotic/eukaryotic taxa (Willms et al. 2021). This contrasts with verrucomicrobial endosymbionts belonging to *Candidatus Xiphinematobacter* (candidate genus *Xiphinematobacter*, family Xiphinematobacteraceae) (Willms et al. 2021) detected as an indicator in transition zones of Erie. *Candidatus_Udaeobacter* is generally highly abundant in low pH soils and can drastically decrease in abundance as soil pH increases above 7. Given its relatively small genome size (∼2.81–3.67 Mbp) compared to other common soil bacteria, minimizing the metabolic expense of synthesizing costly amino acids—which can be acquired from lysed cells in the environment—may offer an advantage in coastal ecosystems (Willms et al. 2021). *Methylocystis* spp. (wetland indicator in Erie) is a methanotrophic bacterium which reduces methane gas fluxes from wetland soils (Oshkin et al. 2020) while *Mycobacterium* spp. (upland indicator in Chesapeake and Erie) has been linked to nitrogen metabolism in coastal soils (Semenova et al. 2022). *Sva0081_sediment_Group* spp. (wetland indicator in Chesapeake) is a known sulfate reducer and H_2_ scavenger (Fonseca et al. 2022) while *Flavobacterium* spp. (wetland indicator in Chesapeake) is known to contain potential nitrate and nitrite reductase genes (Abdelhamed et al. 2021). Taken together, these indicators largely encompass microbial species that are critical in carbon, nitrogen, and sulfur cycling.

We found unique indicator species specific to transect zones (ecosystem states) along the TAIs of Chesapeake and Erie. This reflects the distinct habitats across the TAI gradient shaped by vegetation type, flood duration, and intensity. The indicator OTUs could potentially serve as predictors of an ecosystem state (Paerl et al. 2003, Urakawa and Bernhard 2017, Ma et al. 2022, Ramljak et al. 2024) as a coastal system progresses from an upland to transition or transition to a wetland zone. On the other hand, shared indicators between upland-transition and transition-wetland zones could point towards either a spatial gradient or an ongoing system change. Expanding studies across other coastal TAI gradients will improve the understanding of the relative importance of these indicator species and will help determine universal biomarkers of ecosystem states in coastal ecosystems (Liang et al. 2023).

Universal ecosystem biomarkers for coastal gradients can bridge upland forests, transition zones, and wetlands across diverse geographical locations and environments. Standardized molecular level indicators that track carbon pools, nutrient cycling, and redox-sensitive microbial communities across varying hydrological regimes are a great metric to develop these markers. Studies that build upon SOM, and microbial community characteristics, can create a consistent scalable framework towards this goal. For example, standardized measurements of lignin, lipids, and nitrogen compounds can help track terrestrial inputs into coastal ecosystems (Hedges and Mann 1979, Bianchi 2007). Microbial communities change consistently with elevation, soil chemistry (Fierer et al. 2009) and water saturation such that fungal or plant-derived biomarkers and bacterial taxa such as rhizobium, and actinomycetes can be reliable biomarkers for unsaturated upland soils while sulfate reducing bacteria are typical indicators of anoxic and submerged wetlands (Jørgensen 1982). Data across multiple sites, for example, the EXCHANGE Consortium (Myers-Pigg et al. 2023), are necessary to calibrate biomarkers across different regions ensuring that indicators work across freshwater and salt water influenced ecosystems. Furthermore, biomarker patterns are often influenced by local hydrology implying that they must be calibrated and refined for seasonal or event-based changes such as high and low tides, and hurricanes (Goni et al. 2007). While our study uses DNA-based markers, a combination of DNA, RNA and lipid profiles will enable capture of active communities essential to track when and under what conditions specific functions are being expressed (Qi et al. 2025).

Archaeal indicator taxa are equally important in coastal ecosystems due to their extensive role in methane cycling. Among these, methanogens and methanotrophs are particularly important in wetlands (Urakawa and Bernhard 2017). Estimates from the Global Methane Budget 2000–2020 via bottom-up approaches suggest that wetlands contribute to around 25% of the total (combining natural and anthropogenic) atmospheric methane sources (Saunois et al. 2024). We were unable to conduct in-depth statistical analyses to shed light on archaeal indicator taxa in this study due to low archaeal read abundance, and we acknowledge this as a limitation of the study. This limitation is likely due to a methodological constraint, i.e. the lack of specificity of the universal primer pair 515F-806R for archaea, leading to the preferential amplification of bacterial DNA (Raymann et al. 2017).

### Shared microbiome composition

By identifying a core microbiome that is consistently detected across all transect positions irrespective of site, we highlight important microbial functional groups that are conserved across the TAIs in a region (H1). Core microbiome members are taxa shared between microbial communities and can reflect functional relationships with an environmental system (Shade and Handelsman 2012). To characterize the core microbiome in the coastal TAIs of Erie and Chesapeake, we utilized an abundance-occupancy approach which includes a threshold of taxon proportional contribution (abundance) and minimal detection across samples (occupancy) (Shade and Stopnisek 2019).

The motivation behind the core community analysis was the fact that the abundance-weighted indicator species analysis did not reveal a shared community across all upland, transition and wetland zones in both Chesapeake and Erie. When we relaxed the abundance (0.01%) and prioritized prevalence, we indeed found members that were part of a strict core across both regions. These taxa passed two conditions—they were present in >80% of all samples in the region and they also exceeded or met the abundance threshold in >80% of all samples in the region. Presence of rhizobia and *Geobacter* in the strict core of Erie suggests potential symbiotic nitrogen fixation processes and iron reduction as conserved functions across transect positions and sites in the freshwater TAIs. Among the strict core members of Chesapeake, *Acidothermus* sp. is a cellulolytic actinobacteria, and their genomes have revealed a diverse repertoire of biomass degrading enzymes (Barabote et al. 2009), *Roseiarcus* spp., is an alphaproteobacterial methanotroph (Kulichevskaya et al. 2014) and have potential for fermentative activities utilizing compounds such as methanol and methane (Penna et al. 2025) whereas *Haliangium* spp., is a halotolerant genus (X. Qi et al. 2025). In both Erie and Chesapeake, the presence of *Pseudolabrys* sp. in the strict core suggests metabolic capabilities associated with N and S cycling (Vineis et al. 2025) and has been detected in core communities across riverine, estuarine and coastal sediments across varying environmental conditions (Selvarajan et al. 2024). Together these are valuable targets that suggest potential functional redundancy across the TAIs in both regions. These results also probe the idea that even though microbial community structures are distinct across ecosystem states, some functions may be resilient to environmental change.

Notably, the number of core members detected in Chesapeake were fewer than in Erie. This could be attributed to the salinity gradient in Chesapeake across the three sites imposing a physiological limitation in the number of taxa capable of thriving across varying salinity levels. Further studies that explore a persistent or a dynamic core microbiome that shed light on microbial taxa that persist across major environmental transitions (Neu et al. 2021a) (saltwater intrusion in freshwater systems) are needed to understand temporal dynamics of the core members. This has implications for understanding biogeochemical functions that are most sensitive and robust to environmental change and may include processes relevant to critical coastal ecosystem services such as methane, carbon, sulfur, nitrogen, and iron cycling. While previous studies have used minimum abundance thresholds of 0.001%–4.5% to designate a core member (Neu et al. 2021a), we used a detection limit of 0.01% which was less conservative. Another aspect that can be considered in future work is to combine an abundance-occupancy relationship with the contribution to Bray–Curtis similarity between samples to prioritize core members (Shade and Stopnisek 2019). Furthermore, it has been shown that the core microbiome within microbial clades could reveal different patterns of abundance-occupancy across space (Neu et al. 2021a, 2021b, 2021c).

While we found evidence of core microbes investigated in marine planktons and grasses (Blais et al. 2022, Krabberød et al. 2022, Ugarelli et al. 2024), we found very few studies that investigate and compare core communities from both freshwater and estuarine TAIs using standardized sampling techniques (Selvarajan et al. 2024). Other studies, for example in alpine permafrost systems, have used occupancy-specificity (average abundance within all samples) relationships to look at specialist species that show high abundance and occupancy. One such study (Kang et al. 2024) used a depth-resolved approach to show that specialist species in the surface layer were mostly α/γ-Proteobacteria and Actinobacteria, with Bacteroidia, γ-Proteobacteria, and Actinobacteriota being both specific and common in subsurface layers. While we did not interrogate depth-resolved trends in this study, we investigated the abundance-occupancy relationship at the bacterial class level within each region and found evidence of classes which were high in abundance and high in occupancy (α/γ-Proteobacteria) across all samples. However, there were several instances of classes which were low abundance but very high occupancy (∼1) indicating rare members. These include classes Bacteroidia, Gemmatimonadetes, and Acidimicrobiia in Chesapeake and Acidobacteriae and Desulfuromonadia in Erie. If and how rare community members that are persistently detected along the TAI shift in their competitiveness and activity dynamics is crucial to evaluate as coastal TAIs continue to be impacted by sea level rise and storm surges.

### Correlation networks integrate OTUs and OM features

While characterization of the coastal soil microbiome provides important insights for hypothesis generation and overall functional potential, deciphering coordinated patterns in microbiome and metabolome assembly provides an integrated view of microbiome structure and function. Here, we used ecologically defined indices to test the hypothesis that certain bacterial OTUs, and OM features would have positive relationships in their contribution to homogenous/variable selection of their respective communities within a transect position with implications for OM decomposition (H3).

We emphasize that we refer to OM composition as analogous to metabolomes to be in consensus with terminology published in the literature (Danczak et al. 2020, 2022, Freire-Zapata et al. 2024) and the concepts of which we use to establish this study. However, we acknowledge the difference between the methods used for the two analyses (FTICR-MS for OM composition and LC–MS for metabolomics). As such, the assembly processes that determine the composition of metabolite assemblages can be considered analogous to the community assembly processes (homogenous/variable selection) that govern the composition of ecological metacommunities (Danczak et al. 2020). We use the word “assembly” here in the context of OM (metabolome) formation to emphasize that OM is essentially built via recruitment of organic molecules similar to how a microbial community is assembled from individual microbial species.

We performed the network analysis using both OM and microbial (OTU) features to understand ecological assembly processes and test H3. We emphasize that not all modules show strong linkages between OTUs and OM features (Fig. 4, edge weights based on TOM similarity index). This is an important finding because the weak associations suggest that certain OTUs and OM molecules are less consequential in their coordinated contributions to community assembly. However, Erie wetlands showed strong concerted contributions of several key microbial taxa to community assembly compared to the other transect positions. This suggests that certain biogeochemical functions in wetlands could be tightly linked to specific members of the community, giving us targets to probe in future studies. Coupled with this, a complete loss of OM features in the Erie wetland modules suggest a diminishing trend in coordinated contributions of OTUs and OM features to community assembly along the upland to wetland gradient. We also note that several microbial features in our transect-specific modules are part of the indicator taxa pool which further validates the importance of these keystone species (Table S3).

We found that several indicator nitrogen fixers (Order Rhizobiales and Frankiales), and unsaturated/lignin-like and aromatic compounds similarly contributed to community assembly in Chesapeake upland and Erie transition while indicator nitrogen fixer (Order Rhizobiales), indicator sulfate reducer (Order Desulfobacterales), and condensed aromatics showed coordinated impacts in Chesapeake wetlands. These patterns suggest that certain microbial taxa may impact OM decomposition. Since degradation of lignin and aromatics require oxidative enzymes (Wang et al. 2018), our results point towards these processes in the relatively oxic upland and transition zones of Chesapeake and Erie but a lack of a generic pattern in wetlands; we found no strong associations between lignin/aromatics and microbes in Erie wetlands. Nitrogen fixers such as rhizobia, which were common in the network modules across the TAI positions, are known to degrade aromatic polyphenol compounds such as catechol (Hussien et al. 1974) suggesting possible degradation of similar compounds. Aromatic phenolic compounds such as flavonoids secreted by legumes are often signals in the establishment of legume-rhizobia symbiosis and can help in plant defense processes (Lone et al. 2024) suggesting similar interconnected functional relationships in the uplands and transition zones. Overall, the microbial and OM features that show coordinated impacts on community assembly provide experimental targets to prioritize in future studies.

Previous studies have used null model and neutral community model analyses to provide insights on ecological assembly, such as the importance of stochastic processes and homogeneous selection across coastal wetlands (Gao et al. 2020, 2021) and managed agroecosystems (Ohigashi et al. 2025). However, these studies did not investigate the feature-level contribution to such assembly processes and lacked an integration of microbiome and metabolome datasets. Using such an integrated approach, information about microbial and OM features that similarly contribute to community convergence (homogenous selection) or divergence (variable selection) can be obtained. Specifically, the features in our modules reflect a significant contribution to community convergence within transects (βNTI <−2). This suggests homogenous selection due to consistent environmental pressures across different samples within a transect, leading to less phylogenetic turnover than expected by chance (Stegen et al. 2012). However, it is beyond the scope of this work to specifically demonstrate whether these compounds are utilized or are by-products of specific microbial processes. We also found several modules in our analyses that solely contained OM features, which points to further questions about whether these modules indicate one or several ecological pathways. While a direct answer to these questions mandates more experimentation and genome-resolved approaches, studying overall ecology-function linkages identifies potential associations between OM classes and microbial groups that can be prioritized as targets for future hypothesis generation and experiments.

## Conclusions

Our goal was to determine the common microbial features that link the TAI irrespective of the spatial effects across TAI zones within a region, as well as differentiating features that highlight the key differences between Lake Erie and Chesapeake Bay coastal regions. We found that each region had a distinct microbial community with transects explaining a greater percentage of the variation in the community compared to sites in both regions. While indicator taxa unique to a transect position defined the ecosystem states along the TAI, several indicator taxa were also shared between upland-transition and transition-wetland zones suggesting that the presence of certain microbes was less predictive of the transition zone ecosystem state. Transitions reflected a midpoint when using metrics such as richness and diversity and revealed a linear trend from upland to the wetland state, contrary to expectations given the dynamic hydrology of the transition zone. Core communities within the freshwater Erie region revealed iron and nitrogen cycling microbial functional groups to be conserved along the TAI. This suggests that even though transects differ in microbiome community structure, there are likely certain functional redundancies across them. Coupling the βNTI_feat_ metric across all microbiome and OM features revealed that rhizobia, sulfate reducers, lignin-like and aromatic compounds had coordinated contributions to community assembly in wetlands of Chesapeake. In Erie wetlands, we found strong linkages between several bacterial OTUs but not between OTUs and OM features. By using these microbiome-metabolite linkages we propose important bacterial taxa and organic molecules that could have coordinated roles in metabolic pathways and functions and define targets of interest to generate testable hypotheses for future work.

## Supplementary Material

fiag066_Supplemental_Files

## Data Availability

All raw and processed data are publicly available at NCBI Sequence Read Archive (PRJNA 1345432) and ESS-DIVE (Bandopadhyay et al. 2025, DOI: 10.15485/2589181 accessed via https://data.ess-dive.lbl.gov/datasets/doi:10.15485/2589181). All code used for analysis and visualization is available on GitHub https://github.com/COMPASS-DOE/SynopticSite_16S_FTICR.git (Zenodo, Bandopadhyay 2026, DOI: 10.5281/zenodo.21251201 accessed via https://doi.org/10.5281/zenodo.21251201). Raw FTICR-MS data can be downloaded from the Science Central portal that hosts EMSL data here sc.emsl.pnnl.gov by providing the proposal ID 60602.
