# Supplementary material for: Soil microbial ecology and microbiome-metabolite linkages improve understanding of ecosystem states along terrestrial-aquatic interfaces
Source: FEMS Microbiol Ecol. 2026 Jun 24;102(7):fiag066. doi: 10.1093/femsec/fiag066 (PMC13348248; doi:10.1093/femsec/fiag066)
Supplement: fiag066_Supplemental_Files [file fiag066_supplemental_files.zip › Supplementary_Information_Cleaned_R2_Final_07.01.26.pdf]

Supplementary Information

Soil microbial ecology and microbiome-metabolite linkages improve understanding of ecosystem states along terrestrial-aquatic interfaces

Sreejata Bandopadhyay[1*], Robert E. Danczak[1], Kaizad F. Patel[1], Kathleen R. Beilsmith[2], Pamela B. Weisenhorn[3], Trisha L. Spanbauer[4,5], Nicholas J. Reichart[1], Michael N. Weintraub[4,1], Vanessa L. Bailey[1]

[1]Biological Sciences Division, Pacific Northwest National Laboratory, Richland, WA 99352, USA

[2]Data Science and Learning Division, Argonne National Laboratory, Lemont, IL 60439, USA

[3]Biosciences Division, Argonne National Laboratory, Lemont, IL 60439, USA

[4] Department of Environmental Sciences, University of Toledo, Toledo, OH 43606, USA

[5]Department of Earth and Environmental Sciences, University of Kentucky, Lexington, KY 40506

*Correspondence to sreejata.bandopadhyay@pnnl.gov, Biological Sciences Division, Pacific Northwest National Laboratory, 902 Battelle Boulevard, Richland, WA 99352, USA

Materials and Methods

OTU versus ASV choice

There are several reasons we chose to use OTUs instead of ASVs for this study. First is the problem with signal dilution often observed when using ASVs. Certain analysis such as the indicspecies test in R relies heavily on fidelity - the probability that a microbe is present in all samples of the target group (e.g. transect in our case). ASVs provide high resolution at the single nucleotide level. This high resolution can split an ecologically single population into many separate groups or variants. Consequently, the fidelity of the test being run drastically reduces, by splitting a specific microbial population into several groups/variants each of which have low fidelity, thus leading to failed statistical tests. This means that while a specific microbial functional group (e.g. *Bacillus subtilis*) might be present across all samples and be an important indicator for a specific transect, it will not show up as an indicator merely due to its presence being split across ASVs. Second, environmental gradients often select for specific microbial functional groups rather than specific strains of functional groups that differ by few nucleotides. This signal is therefore likely to be captured better and with statistical significance at an agglomerated OTU level versus the ASV. Third, grouping ASVs into OTUs at 97-99% similarity usually collapses intragenomic variants (multiple copies of 16S rRNA gene within a single genome each with differing nucleotides) into a single group. Finally, in highly complex environmental matrices like soil, there is high spatial heterogeneity and finding the exact same strain (ASV) across all samples in a specific group is rare. Thus, an OTU clustered at 97-99% similarity has better chances of appearing in more samples of that group as compared to an ASV, hence crossing the threshold for significance in fidelity for most ecological tests, a signal that will largely be missed when using ASVs.

Indicator species and core microbiome analysis

We used the indicspecies package in R (Chytrý et al., 2002) to find the indicator OTUs associated with upland, transition and wetland zones across the coastal TAIs of Chesapeake and Erie. Within the package, a taxon is defined as an indicator if it meets two criteria:

1. Exclusivity or specificity: The taxon is found in one group and not others (for example: only in upland, and not in wetland)
2. Consistency/fidelity: The taxon is found in most samples of the group.

In an ecological context, we can then predict or "indicate" the group identity. If we find an indicator taxon in an unknown sample from a site, we can predict with high probability which group (in our case transect position) that sample belongs to. There are different indices that are used in community ecology studies to find indicator taxa such as the Indicator Value (IndVal) index used to measure the association between a species and a site group. The statistical significance of the relationship is tested using a permutation test. However, the index we use to analyze the association between a species and a group of transects is a statistic focused on fidelity, and distinct from the IndVal component. This metric is Pearson's phi coefficient of association. While this coefficient can be calculated on a presence-absence dataset, we chose to use abundance values instead. The abundance-based counterpart of the phi coefficient is called the point biserial correlation coefficient which is the metric used to determine the indicator taxa associated with upland, transition and wetland zones in our study.

The core microbiome analysis can be used to denote taxa at genus, species, or other taxonomic levels. However, we specifically chose to do this only at the genus level (Dueholm et al., 2022) because the V4 region of the 16S rRNA gene lacks enough unique genetic variation to distinguish between closely related species. Across samples within a region, we also looked at

the conditionally rare and abundant (CRAT) genera which include taxa that are more than 1% abundant in at least one sample but are not present in any of the core designations. CRAT taxa are generally low in abundance but can become prevalent due to environmental disturbances (Dueholm et al., 2022). In a coastal system, these can be processes that dominate in the immediate aftermath of water inundation along a coastal transect, including water table rise due to fluctuations in precipitation levels or similar.

Archaeal community characterization

We characterized the archaeal domain to look for methanogens in the community. However, the total read counts of the archaea were substantially lower than bacteria across samples (the highest read count was around 7000 reads for only select samples). Most of the samples had less than 3000 reads. Since archaea represent a different branch in the tree of life, we chose to analyze the archaeal community separately from the bacterial community. We normalized to 2000 reads across samples to effectively compare archaeal abundances. We report the dominant members of the archaeal community separately from the bacterial community along with the beta diversity of archaea across Chesapeake and Erie.

Fourier-transform ion cyclotron resonance mass spectrometry (FTICR-MS)

Soil organic carbon was extracted as described in (Patel et al., 2021) with slight modifications. Briefly, extractions were done with Milli-Q deionized water (1:10 w/w) by shaking at 150 rpm at room temperature for 1 h, centrifuging at 7000 g for 20 min, and then filtering through 0.45 μm polyethersulfone (PES) filters. The water-extractable organic carbon was characterized by electrospray ionization (ESI) coupled with Fourier-transform ion cyclotron resonance mass spectrometry (FTICR-MS) located at the Environmental Molecular Sciences Laboratory

(EMSL), a DOE user facility located in Richland WA. Water extracts were desalted and concentrated by solid phase extraction using PPL cartridges (Agilent, Santa Clara, CA, USA) (Dittmar et al., 2008) and were eluted in methanol before direct injection on a 12 T Bruker SolariX FT-ICR-MS spectrometer; see (Tfaily et al., 2015, 2017) for detailed methods. Ninety-six individual scans were averaged for each sample and internally calibrated using organic matter (OM) homologous series separated by 14 Da (–CH2 groups). The mass measurement accuracy was less than 1 ppm for singly charged ions across a broad m/z range (i.e., 200, < m/z < 1200). Chemical formulae were assigned using the Formultitude (previously named Formularity) software (https://github.com/PNNL-Comp-Mass-Spec/Formultitude) , including only peaks with a signal/noise ratio >7 (Kujawinski and Behn, 2006; Tolić et al., 2017), with the restrictions C_{1-130}, H_{1-200}, O_{1-50}, N_{0-3}, S_{0-3}, P_{0-2}.

Further processing was done using the *fticrrr* package in R (Patel, 2020). FTICR-MS-resolved peaks were analyzed only on a presence/absence basis, due to established issues with intensities and ionization efficiencies in complex matrices using ESI (Kujawinski, 2002; Ohno et al., 2016; Kew et al., 2024). Only peaks identified in ≥2/3 of the replicates were considered present within that treatment (Payne et al., 2009; Sleighter et al., 2012). The modified Aromaticity Index (AI_{mod}), used to classify identified molecules, was calculated from (Koch and Dittmar, 2016). The identified peaks were assigned to one of the following classes following the method of (Seidel et al., 2014, 2017) using the AI_{mod} and molecular H:C and O:C ratios: (1) polycyclic condensed aromatics ($AI_{mod} > 0.66$); (2) highly aromatic compounds, which include polyphenols and polycyclic aromatic compounds with aliphatic chains ($0.66 > AI_{mod} > 0.50$); (3) highly unsaturated compounds, which include phenols such as soil-derived products of lignin

degradation ($AI_{mod} \leq 0.50$ and $H/C < 1.5$); and (4) aliphatic compounds ($H/C \geq 1.5$), including unsaturated aliphatics, N-containing aliphatics, and saturated compounds including fatty and sulfonic acids, and/or carbohydrates. The FT-ICR-MS compound classes are tentative classifications as they are solely based on the indices (O/C and H/C ratios and AI_{mod} values) from the molecular formula, not the molecular structure. We generated a molecular characteristics dendrogram for dendrogram-informed analysis using the methods outlined in (Danczak et al., 2020) for βNTI_{feat} calculations as described below.

Weighted gene co-expression network analysis

We used the default settings in WGCNA for the analyses. Briefly, a soft thresholding power was selected to build networks, a key feature of WGCNA that assigns a connection weight to each correlated gene pair (Ko and Brandizzi, 2023). The function "pickSoftThreshold" was used to identify this power value that fits the dataset based on the criteria for approximate scale-free topology. The coefficient of determination (R^2 value, where 1=perfectly linear relationship) was used to evaluate the scale-free topology model fit. We chose a soft-thresholding power based on the lowest power value that passed a reasonably high R^2 cutoff value (~0.8) and resided on the scale-free topology fit index curve. Next, we calculated the adjacency matrix from the correlations and the soft thresholding power and finally the TOM (topology overlap matrix) similarity from the adjacency matrix. Then, features were assigned to modules using average linkage hierarchical clustering using the blockwiseModules function within the WGCNA package (Zhang and Horvath, 2005). For each module, we calculated the R^2 value for each feature's correlation with the module eigenvalue. Network-specific stats included estimates of closeness, betweenness, degree, and hub score (Table 6, Supplementary Data). For visualization

we did not set any TOM threshold, to retain all features assigned to a module. For all network statistics, we set the TOM threshold to greater than 0.1.

Results

Microbial sequencing data summary

For bacterial communities, all samples had adequate coverage for read depth with most samples having a read count greater than 30,000 (**Fig. S1C**). As per the rarefaction curves, we rarefied samples to 20,000 reads for both the Chesapeake and Erie regions. This led to a loss of 6 samples from the total of 205 (1 from Chesapeake and 5 from Erie) that did not meet the rarefaction threshold. All analyses were performed on a total of 199 samples (117 from Chesapeake and 82 from Erie, sample breakdown across transects and sites are in Table 1, Supplementary Data). Overall DNA concentrations were higher for the Erie samples as compared to the Chesapeake samples which could be attributed to the different extraction kits and protocols used in the extractions (Tables 2-3, Supplementary Data). However, we rarefied all samples to even depth to make sure to limit any bias that may have occurred due to higher DNA yields from Erie samples. The archaeal community was analyzed separately to account for equal sequencing depth of archaea across samples. Overall, most samples had very low archaeal read counts with the majority having less than 3000 reads. We rarefied reads across all samples to 2000 read counts to comparatively assess the archaeal domain's community composition (**Fig. S2 A, B, C**). However, this led to a loss of 131 samples, with only 74 samples retained across Chesapeake (22 samples) and Erie (52 samples).

Archaeal community across regions and transects

Similar to the bacterial community, the archaeal community differed significantly between regions (Pseudo-F=78.44, P=0.001) with significant interaction between transect:region (Pseudo-F=16.29, P=0.001) and site:transect (F=2.69, P=0.002). Within each region, significant differences were observed between site (Pseudo-F=13.24, P=0.001 for Chesapeake, Pseudo-F= 8.62, P=0.001 for Erie) and transect (Pseudo-F=9.18, P=0.001 for Chesapeake, Pseudo-F=30.66, P=0.001 for Erie) (**Fig. S4 A, B, C**). Within the archaeal domain, we found several methanogens and ammonia-oxidizing archaea across Chesapeake and Erie. For instance, members of the *Nitrosphaeraceae* family containing ammonia-oxidizing archaea (AOA) were found in high abundance across upland, transition, and wetland zones in Erie but were not found in Chesapeake. Similarly, other AOA such as *Candidatus nitrocosmicus* (candidate genus *Nitrocosmicus*, family *Nitrososphaeraceae*) were found in the upland and transition zones of Erie but had low abundance in Chesapeake. This is consistent with high nitrate concentrations reported in Erie sites compared to Chesapeake (Patel et al., 2025). However, within Chesapeake upland and transition zones, we also found instances of AOA, which were not seen in Erie; for example, members of class Nitrososphaeria. Methylotrophs, such as those belonging to Bathyarchaeia, were observed in high abundance in wetlands of Chesapeake and Erie (**Fig. S4D**). Among archaeal methanogens, we found *Methanobacterium* and *Methanothrix* in wetland Erie, suggesting the importance of methane production in coastal wetlands and implications for methane cycling.

Table S1: Site characteristics and soil properties for **A.** Chesapeake Bay and **B.** Lake Erie. C. A subset of the soil chemical analytes measured at the sites in Chesapeake and Erie. The soil chemistry data was used to understand the biogeochemical drivers of microbial community structure (Fig. 3). Data in Table C are presented as mean ± standard error for each site, followed by a mean across all sites for a given transect position. Different lower-case letters denote statistically significant differences among sites for a given transect position. Different upper-case letters denote statistically significant differences among transect positions for a given region. Different Greek letters denote significant differences among transect positions for a given site.

**A.**	**Global Change Research Wetland (GCW)**			**Moneystump Marsh (MSM)**			**Goodwin Islands (GWI)**		
	**Upland**	**Transition**	**Wetland**	**Upland**	**Transition**	**Wetland**	**Upland**	**Transition**	**Wetland**
**Lat (N)**	38.874445	38.874440	38.874948	38.43085	38.43042	38.42923	37.21927	37.21928	37.21892
**Lon (E)**	-76.551667	-76.551110	-76.549995	-76.22622	-76.22708	-76.22739	-76.40851	-76.40937	-76.41002
**Elevation (m)**	7.09	1.21	0.24	0.68	0.48	0.29	1.03	0.79	0.68
**Salinity of surface water (psu)**	5-10			5-10			18-25		
**MAT** (°C)[1]	14.03			14.03			17.53		

**MAP (cm)**	126.8			126.8			141.8		
**Vegetation**	Mixed deciduous tree community including *Acer rubrum* (red maple), *Fagus grandifolia* (American beech) and *Quercus alba/falcata* (white oak/southern red oak)	Mixed deciduous tree community including *Acer rubrum, Fagus grandifolia* and *Quercus alba/falcata*	Tidal marsh dominated by *Spartina patens* (saltmeadow cordgrass), *Distichlis spicata* (desert saltgrass)	*Pinus taeda* (loblolly pine) conifer forest	*Pinus taeda* conifer forest	Salt marsh dominated by *Spartina patens, Distichlis spicata* and *Schoenoplectus robustus*	*Pinus taeda* forest	*Pinus taeda* forest with a substantial *Phragmites australis* understory	Salt marsh dominated by *Spartina patens, Distichlis spicata*
Parent material[2]	Glauconite-bearing loamy fluviomarine deposits	Loamy alluvium	Herbaceous plant material over marine coastal deposits	Loamy Fluvio-Deltaic Coastal Sediments	Clayey Fluvio-Deltaic Coastal Sediments	Clayey Fluvio-Deltaic Coastal Sediments	Marine deposits	Marine deposits	Marine deposits
Soil Series	Collington and Annapolis, fine sandy loam (Typic Hapludults)	Widewater and Issue, silt loam (Fluvaquentic Endoaquept and Dystrudeptss)	Mispillon and Transquaking, mucky peat	Elkton mucky silt loam (Typic Endoaquults)	Sunken mucky silt loam, occasionally flooded, tidal (Typic Endoaqualfs)	Honga peat, very frequently flooded, tidal (Terric Sulfihemists)	Dragston fine sandy loam (Aeric Endoaquults)	Nimmo fine sandy loam (Typic Endoaquults)	Axis fine sandy loam (Typic Sulfaquents)
Texture									
Sand (%)	47.27 ± 2.31	40.78 ± 9.31	–	–	–	–	–	–	–
Silt (%)	36.7 ± 3.6	32.16 ± 4.95	–	–	–	–	–	–	–

Clay (%)	16.04 ± 2.41	27.07 ± 4.36	–	–	–	–	–	–	–
Texture class	*Loam*	*Clay loam*	*Organic*	*Organic*	*Organic*	*Organic*	*Organic*	*Organic*	*Organic*

**B.**	**Crane Creek (CRC)**			**Portage River (PTR)**			**Old Woman Creek (OWC)**		
	**Upland**	**Transition**	**Wetland**	**Upland**	**Transition**	**Wetland**	**Upland**	**Transition**	**Wetland**
**Lat (N)**	41.61524	41.62192	41.62185	41.50148	41.502723	41.50174	41.37618	41.376017	41.376445
**Lon (E)**	-83.22889	-83.23815	-83.2389	-83.04611	-83.045662	-83.04372	-82.50685	-82.507507	-82.509024
**Elevation (m)**	175.2	175.1	175.1	175.7	175.4	174.9	179.7	175.2	174.8
**MAT (°C)[1]**	10.64			10.64			10.64		
**MAP (cm)**	113.2			113.2			113.2		
**Vegetation**	Mixed broadleaf forest dominated by *Quercus alba* (white oak)	Mixed broadleaf forest dominated by *Quercus bicolor* (swamp white oak)	Freshwater emergent marsh dominated by fragmented zones of *Typha latifolia, Peltandra virginica* (green arrow arum),	Mixed broadleaf forest dominated by *Carya ovata* (shagbark hickory)	Mixed broadleaf forest dominated by *Carya ovata*	Freshwater emergent marsh dominated by fragmented zones of *Typha latifolia* (bulrush), *Phragmites australis,* and *Phalaris*	Mixed broadleaf forest dominated by *Quercus rubra* (northern red oak)	Mixed broadleaf forest dominated by *Quercus rubra*	Freshwater emergent marsh dominated by fragmented zones of *Saurus cernuus* (lizard's tail) and

			Phragmites australis (common reed), and *Phalaris arundinacea* (reed canary grass)			*arundinacea*			*Phalaris arundinacea*
Parent material[2]	Fine Textured Lacustrine Deposits	Fine Textured Lacustrine Deposits	Fine Textured Lacustrine Deposits	Fine Textured Lacustrine Deposits	Fine Textured Lacustrine Deposits	Fine Textured Lacustrine Deposits	Fine Textured Lacustrine Deposits	Fine Textured Lacustrine Deposits	Fine Textured Lacustrine Deposits
Soil Series	Toledo silty clay and Nappannee silty clay loam (Mollic Endoaquepts and Aeric Epiaqualfs)	Toledo silty clay, flooded (Mollic Endoaquepts)		Toledo silty clay (Mollic Endoaquepts)	Toledo silty clay, flooded (Mollic Endoaquepts)		Zurich, Bixler (Oxyaquic and Aquic Arenic Hapludalfs)	Holly silt loam (Fluvaquentic Endoaquepts)	Fluvaquents, frequently flooded
Texture									
Sand (%)	9.9 ± 1.92	9.44 ± 0.5	4.46 ± 0.58	14.37 ± 0.46	12.73 ± 1.26	6.02 ± 1.1	28.3 ± 1.64	10.69 ± 2.14	3.22 ± 0.28
Silt (%)	69.77 ± 6.02	79.7 ± 5.39	31.74 ± 4.62	83.53 ± 2.41	67.63 ± 9.15	49.08 ± 6.4	57.75 ± 6.82	77.53 ± 9.22	90.3 ± 5.71
Clay (%)	20.33 ± 4.16	10.87 ± 5.04	63.8 ± 4.61	2.1 ± 1.95	19.64 ± 8.02	44.9 ± 7.35	13.96 ± 7.95	11.79 ± 11.37	6.48 ± 5.99
Texture class	*Silt loam*	*Silt loam*	*Clay*	*Silt*	*Silt loam*	*Silty clay*	*Silt loam*	*Silt loam*	*Silt*

[1]Data from https://climatereanalyzer.org/
[2]Data from https://soilexplorer.net/

C.	Site	Erie			Chesapeake		
		Upland	Transition	Wetland	Upland	Transition	Wetland
Sulfate (meq/100 g)	CRC	0.04 ± 0 a*b β*	0.64 ± 0.21 *a β*	6.66 ± 0.93 *a α*	GCW	0.01 ± 0 *b β*	0.13 ± 0.03 *b β*
	PTR	0.05 ± 0.01 *a β*	0.14 ± 0.03 *b β*	2.67 ± 0.67 *b α*	MSM	0.28 ± 0.03 a*b β*	2.17 ± 0.16 *a β*
	OWC	0.03 ± 0 *b β*	0.07 ± 0.01 *b β*	1.87 ± 0.21 *b α*	GWI	0.6 ± 0.2 *a β*	2.07 ± 0.17 *a β*
	Mean	0.04 ± 0 *B*	0.28 ± 0.08 *B*	3.59 ± 0.53 *A*	Mean	0.3 ± 0.08 *B*	1.46 ± 0.21 *B*
NH4-N (µg/g)	CRC	13.11 ± 0.41 *a β*	21.67 ± 0.92 *a α*	23.88 ± 2.69 *a α*	GCW	23.04 ± 1.16 *b α*	10.04 ± 3.79 *b β*
	PTR	11.42 ± 0.44 *a β*	16.3 ± 2.5 a*b β*	25.07 ± 1.75 *a α*	MSM	35.77 ± 2.64 *a β*	76.28 ± 11.55 *a αβ*
	OWC	12.76 ± 0.85 *a β*	13.82 ± 0.7 *b β*	18.96 ± 0.89 *a α*	GWI	22.94 ± 2.01 *b β*	94.56 ± 9.2 *a α*
	Mean	12.43 ± 0.36 C	17.26 ± 1.09 *B*	23.21 ± 1.19 *A*	Mean	27.25 ± 1.68 *B*	60.29 ± 8.99 *AB*
Na (meq/100g)	CRC	0.05 ± 0.01 *a β*	0.34 ± 0.05 *a α*	0.38 ± 0.03 *a α*	GCW	0.04 ± 0 *c β*	0.49 ± 0.16 *c β*
	PTR	0.06 ± 0.01 *a* ψ	0.16 ± 0.02 *b β*	0.38 ± 0.02 *a α*	MSM	5.93 ± 0.44 *b β*	18.75 ± 0.79 *b β*
	OWC	0.03 ± 0 *b ψ*	0.09 ± 0.01 *b β*	0.2 ± 0.01 *b α*	GWI	14.78 ± 1.45 *a β*	39.72 ± 1.24 *a α*
	Mean	0.05 ± 0 C	0.19 ± 0.03 *B*	0.34 ± 0.02 *A*	Mean	6.92 ± 1.35 C	19.65 ± 3.37 *B*
P (µg/g)	CRC	42.06 ± 20.21 *b αβ*	19.21 ± 2.42 *b β*	98.89 ± 27.71 *a α*	GCW	4.7 ± 0.65 *b β*	5.21 ± 0.83 *b β*
	PTR	204.26 ± 13.9 *a α*	256.48 ± 58.33 *a α*	58.02 ± 12.19 *a β*	MSM	3.54 ± 0.85 *b β*	6.24 ± 1.05 *b β*

	OWC	20.92 ± 4.11 *b ψ*	160.87 ± 17.34 *a α*	109.85 ± 9.38 *a β*	GWI	29.24 ± 8.79 *a α*	11.74 ± 1.58 *a α*
	Mean	74.68 ± 17.69 *B*	145.52 ± 27.31 *A*	82.47 ± 10.47 *B*	Mean	12.88 ± 3.89 *B*	7.73 ± 0.89 *B*
CEC (meq/100g)	CRC	14.11 ± 0.46 *a β*	21.8 ± 1.38 *a α*	22.67 ± 1.93 *a α*	GCW	8.61 ± 1.08 *b β*	8.32 ± 0.64 *c β*
	PTR	12.17 ± 0.17 *b ψ*	15.53 ± 1.06 *b β*	20.86 ± 0.61 *a α*	MSM	18.85 ± 4.54 a*b β*	41.92 ± 2.92 *b β*
	OWC	8.1 ± 0.58 *c β*	12.87 ± 0.58 *b α*	12.44 ± 1.24 *b α*	GWI	28.43 ± 2.31 *a β*	86.31 ± 2.66 *a α*
	Mean	11.46 ± 0.55 C	16.73 ± 0.94 *B*	19.26 ± 0.98 *A*	Mean	18.63 ± 2.37 C	45.52 ± 6.78 *B*
Total C, %	CRC	7.14 ± 0.42 *a β*	12.02 ± 0.96 *a α*	7.66 ± 0.33 *a β*	GCW	4.22 ± 0.48 *b ψ*	7.63 ± 0.76 *b β*
	PTR	7.27 ± 0.26 *a α*	10.08 ± 1.87 *ab α*	7.71 ± 0.25 *a α*	MSM	32.64 ± 1.09 *a α*	29.67 ± 1.33 *a α*
	OWC	5.11 ± 0.39 *b α*	6.48 ± 0.46 *b α*	6.5 ± 0.58 *a α*	GWI	32.26 ± 1.67 *a α*	26.27 ± 1.53 *a α*
	Mean	6.51 ± 0.28 *B*	9.64 ± 0.84 *A*	7.39 ± 0.22 *B*	Mean	23.04 ± 2.85 *A*	20 ± 2.42 *A*
Total N, %	CRC	0.52 ± 0.03 *a β*	0.83 ± 0.05 *a α*	0.73 ± 0.04 *a α*	GCW	0.23 ± 0.03 *b ψ*	0.41 ± 0.04 *c β*
	PTR	0.57 ± 0.02 *a α*	0.81 ± 0.15 *a α*	0.68 ± 0.02 *a α*	MSM	1.13 ± 0.07 *a β*	1.52 ± 0.08 *a α*
	OWC	0.36 ± 0.02 *b β*	0.45 ± 0.03 *b αβ*	0.51 ± 0.04 *b α*	GWI	1.26 ± 0.07 *a α*	1.22 ± 0.07 *b α*
	Mean	0.48 ± 0.02 *B*	0.71 ± 0.06 *A*	0.65 ± 0.02 *A*	Mean	0.87 ± 0.1 *B*	1 ± 0.12 *B*
Total S, %	CRC	0.05 ± 0 *a* ψ	0.15 ± 0.01 *a β*	0.21 ± 0.03 *a α*	GCW	0.02 ± 0 *b β*	0.06 ± 0.01 *c β*
	PTR	0.06 ± 0 *a β*	0.09 ± 0.02 *b αβ*	0.13 ± 0.01 *b α*	MSM	0.19 ± 0.05 *a β*	0.43 ± 0.06 *a β*

	OWC	0.03 ± 0 *b β*	0.04 ± 0 *c β*	0.09 ± 0.01 *b α*	GWI	0.15 ± 0.01 *a β*	0.27 ± 0.02 *b αβ*
	Mean	0.05 ± 0 C	0.09 ± 0.01 *B*	0.14 ± 0.01 *A*	Mean	0.12 ± 0.02 *B*	0.22 ± 0.04 *B*
pH	CRC	6.71 ± 0.06 *a α*	6.51 ± 0.11 *ab α*	6.72 ± 0.15 *a α*	GCW	5.22 ± 0.13 *a α*	4.6 ± 0.07 *c β*
	PTR	6.01 ± 0.15 *b α*	6.03 ± 0.26 *b α*	6.57 ± 0.16 *a α*	MSM	4.16 ± 0.08 *b β*	5.18 ± 0.16 *b α*
	OWC	6.2 ± 0.18 *b β*	6.97 ± 0.1 *a α*	6.36 ± 0.1 *a β*	GWI	4.61 ± 0.17 *b ψ*	6.55 ± 0.05 *a α*
	Mean	6.31 ± 0.1 *A*	6.5 ± 0.12 *A*	6.56 ± 0.09 *A*	Mean	4.67 ± 0.12 *B*	5.44 ± 0.18 *A*
Specific Conductance, mS/cm	CRC	0.11 ± 0 a*b β*	0.2 ± 0.02 *a β*	0.63 ± 0.11 *a α*	GCW	0.05 ± 0.03 *b β*	0.05 ± 0.01 *b β*
	PTR	0.12 ± 0.01 *a β*	0.2 ± 0.09 *a αβ*	0.34 ± 0.04 *b α*	MSM	0.61 ± 0.07 *a* ψ	1.79 ± 0.14 *a β*
	OWC	0.08 ± 0.01 *b β*	0.08 ± 0.01 *a β*	0.19 ± 0.02 *b α*	GWI	0.82 ± 0.24 *a β*	1.95 ± 0.13 *a α*
	Mean	0.1 ± 0.01 *B*	0.16 ± 0.03 *B*	0.39 ± 0.05 *A*	Mean	0.49 ± 0.1 *B*	1.26 ± 0.19 *B*
% OM	CRC	15.01 ± 0.82 *a β*	24.82 ± 2.15 *a α*	15.6 ± 1.05 *a β*	GCW	7.7 ± 0.91 *b ψ*	14.04 ± 1.15 *c β*
	PTR	15.05 ± 0.51 *a α*	21.14 ± 4.14 *a α*	17.26 ± 0.54 *a α*	MSM	73.29 ± 4.89 *a α*	75.59 ± 1.68 *a α*
	OWC	10.89 ± 0.76 *b α*	15.44 ± 2.21 *a α*		GWI	64.23 ± 4.09 *a α*	62.47 ± 3.02 *b α*
	Mean	13.65 ± 0.55 *B*	20.47 ± 1.82 *A*	16.64 ± 0.53 *B*	Mean	48.41 ± 6.39 *A*	50.7 ± 5.64 *A*

Table S2: Core microbiome members (at genus level) in **A.** Chesapeake and **B.** Erie that make up the strict core and the general core. Strict and general core members are defined as per (Dueholm et al., 2022) as those taxa that are present in >0.1% abundance in >80% and >50% of the total number of samples respectively.

**A. Chesapeake (selected genera with successful taxonomic assignments at Genus level using SILVA reference database)**		
**Strict Core (> 80 %)**		
Acidothermus<br>*Pseudolabrys*<br>*RCP2-54*	*Roseiarcus*<br>*Mycobacterium*<br>*Aquisphaera*<br>*Bryobacter*	
**General Core (> 50 %)**		
Candidatus_Udaeobacter<br>*Occallatibacter*<br>*Acidibacter*<br>*Candidatus_Koribacter*<br>*Bradyrhizobium*<br>*Conexibacter*	*KD4-96*<br>*Bacillus*<br>*KF-JG30-C25*<br>*Candidatus_Solibacter*<br>*67-14*<br>*WPS-2*	*FCPU426*<br>*ADurb.Bin063-1*<br>*WD2101_soil_group*<br>*Haliangium*<br>*IMCC26256*<br>*BIrii41*<br>*Pedosphaeraceae*

**B. Erie (selected genera with successful taxonomic assignments at Genus level using SILVA reference database)**			
**Strict core (>80%)**			
Candidatus_Udaeobacter	*Haliangium*		
Gaiella	*Rokubacteriales*		
Bacillus	*Pedosphaeraceae*		
Pseudolabrys	*Clostridium_sensu_stricto_1*		
Vicinamibacteraceae	*Clostridium_sensu_stricto_13*		
Candidatus_Xiphinematobacter	*Nitrospira*		
bacteriap25	*Latescibacterota*		
Bradyrhizobium	*Geobacter*		
Mycobacterium	*Chthoniobacter*		
ADurb.Bin063-1			
**General core (>50%)**			
Bacteroidetes_vadinHA17	*Hyphomicrobium*	*Allorhizobium-Neorhizobium-Pararhizobium-Rhizobium*	*Latescibacteraceae*
Acidothermus	*Pedomicrobium*	*Sphingomonas*	*Luedemannella*
Acidibacter	*Pir4_lineage*	*Anaeromyxobacter*	*Terrimonas*
Defluviicoccus	*Paenibacillus*	*Luteolibacter*	*Bryobacter*
Solirubrobacter	*Streptomyces*	*Methyloligellaceae*	*Gemmata*
Methylocystis	*Nocardioides*	*Candidatus_Solibacter*	*Rhodomicrobium*
Conexibacter	*Pirellula*	*Gemmatimonas*	*Reyranella*
Anaerolinea	*Flavobacterium*	*Pseudonocardia*	*Candidatus_Alysiosphaera*
			Cryobacterium
			Phaselicystis

Table S3: Summary of the microbial and organic matter features in the WGCNA modules in Fig. 4.

Region	Transect	Indicator taxa	Module number within transect	Feature (Order level designation for OTUs)
Chesapeake	Upland	Yes	2	Rhizobiales
		NA		Unsaturated/lignin
		NA		Aromatic
		Yes	11	Frankiales
		NA		Unsaturated/lignin

		NA		Aromatic
	Wetland	Yes	2	Rhizobiales
		NA		Unsaturated/lignin
		NA		Aromatic
		NA		Condensed aromatic
		Yes	7	Desulfobacterales
		NA		Unsaturated/lignin
		NA		Aromatic
		NA		Condensed aromatic
Erie	Upland	No	33	Rhizobiales
		NA		Unsaturated/lignin
		NA		Aromatic
		Yes	63	Streptomycetales
		NA		Unsaturated/lignin
		NA		Aromatic
	Transition	Yes	26	Rhizobiales
		NA		Unsaturated/lignin
		NA		Aromatic
		No	8	Clostridiales
		NA		Unsaturated/lignin
	Wetland	Yes	2	Anaerolineales
		Yes		KD4-96
		No		Solirubrobacterales
		Yes		Clostridiales
		No		Peptostreptococcales-Tissierellales

		No		Bacillales
		No		Chthoniobacterales
		Yes		Desulfobacterales
		No		Rhizobiales
		Yes		Ignavibacteriales
		Yes	1	PeM15
		Yes		Anaerolineales
		No		MB-A2-108
		Yes		Solirubrobacterales
		Yes		Pedosphaerales
		Yes		Burkholderiales
		Yes		Vicinamibacterales
		Yes		Desulfobaccales
		Yes		Desulfobacterales
		Yes		Nannocystales
		No		bacteriap25
		Yes		Rhizobiales
		Yes		Bacteroidales

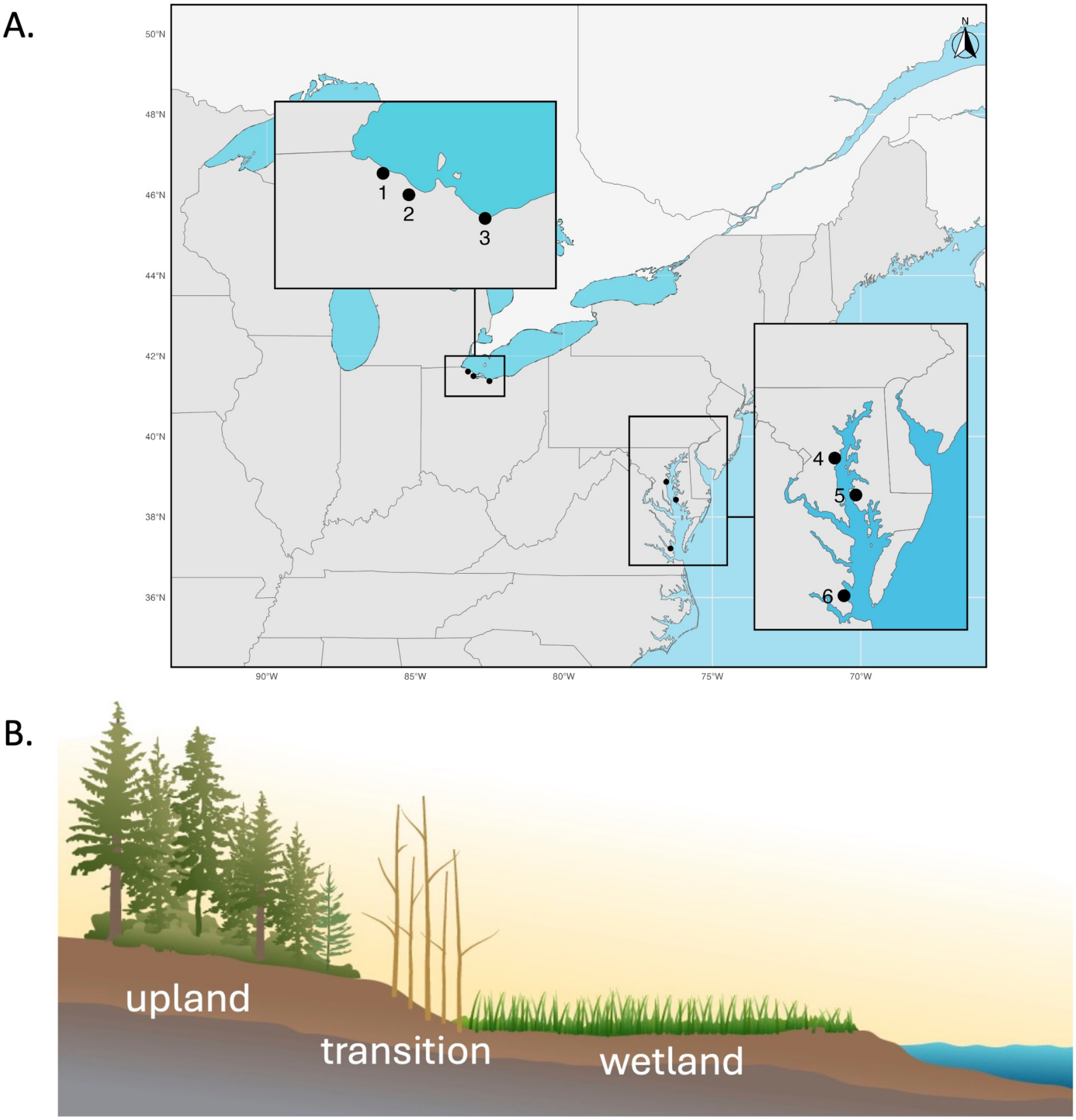

Fig. S1. A. Site location maps. Lake Erie sites: (1) CRC - Crane Creek and (2) PTR - Portage River are in the Western Basin, and (3) OWC - Old Woman Creek is in the Central Basin of Lake Erie. Chesapeake Bay sites: (4) GCW - Global Change Research Wetland, (5) MSM - Moneystump Marsh, and (6) GWI - Goodwin Islands). B. A coastal transect, with upland, transition, and wetland zones. Figure adapted from Patel et. al (2025).

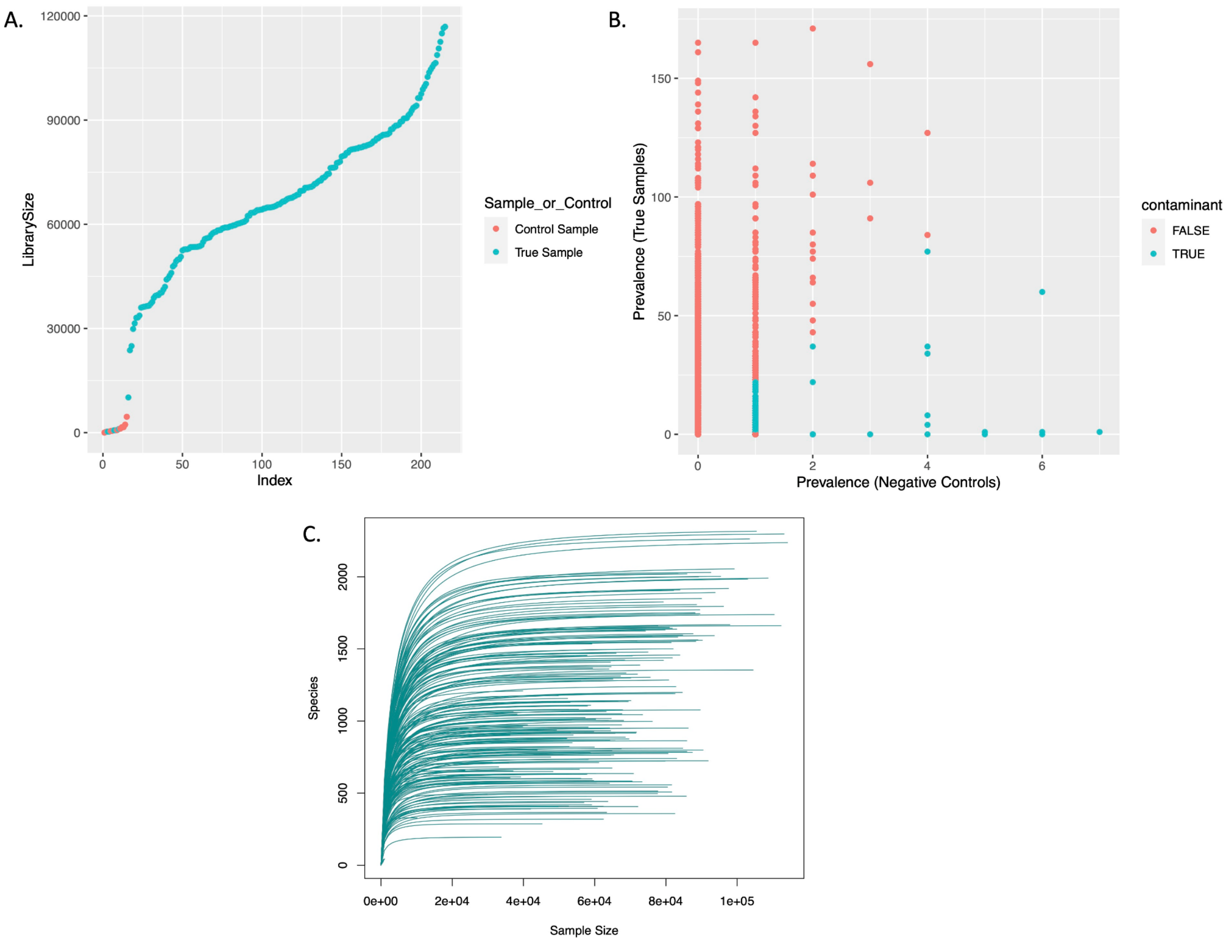

Fig. S2. Library size, prevalence of OTUs across true positive samples and negative controls and rarefaction curves for bacterial communities. **A.** Library size (read counts) of negative controls and samples using package decontam in R. **B.** Prevalence (presence/absence) across samples of a sequence feature (OTU) in true positive samples is compared to the prevalence in negative controls to identify contaminants using package decontam. **C.** Rarefaction curves showing sufficient sequencing coverage for all samples in Chesapeake and Erie. Curves plateau indicating that more sequence reads do not contribute to adding new members to the community. All samples were rarefied to an even read depth of 20,000 reads based on these results.

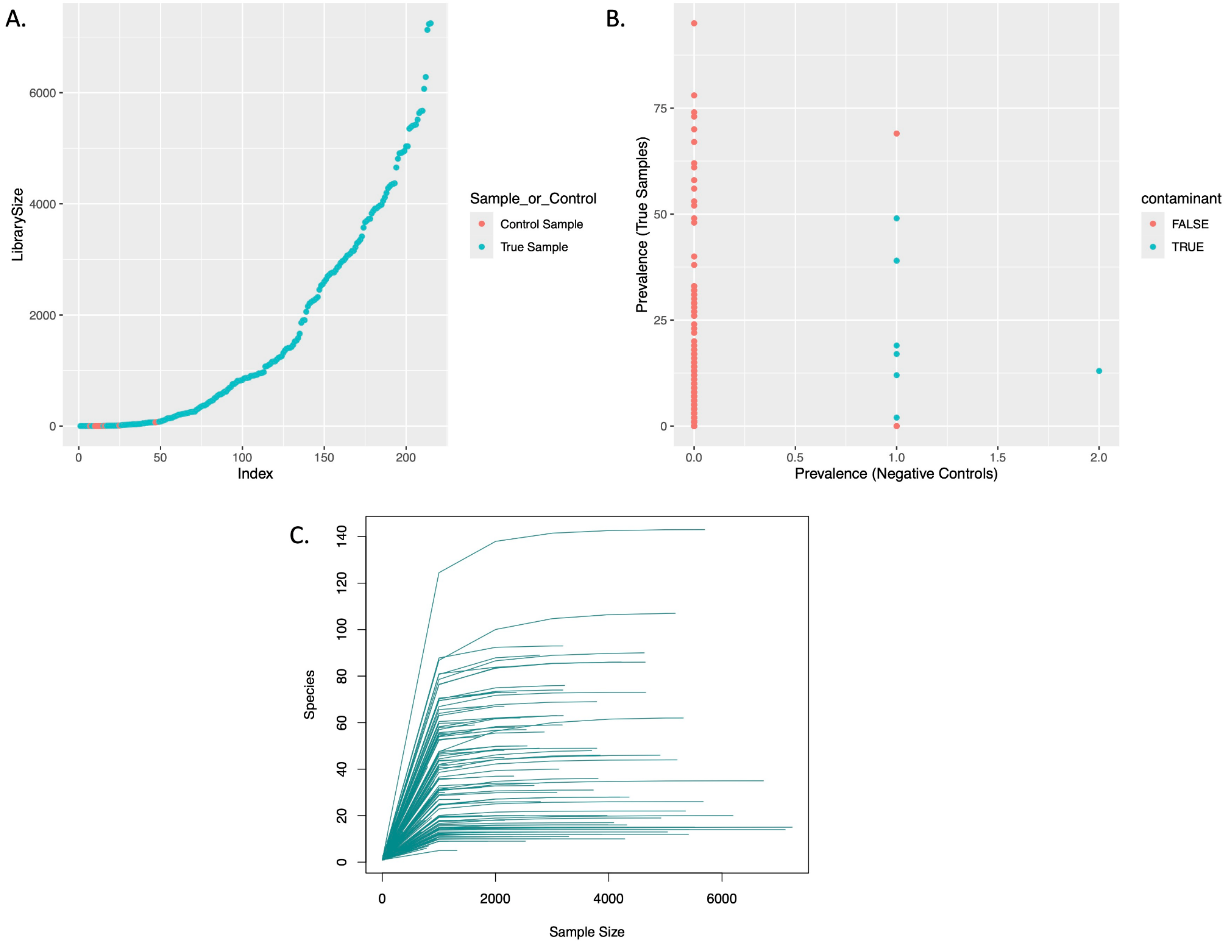

Fig. S3: Library size, prevalence of OTUs across true positive samples and negative controls and rarefaction curves for archaeal communities. **A.** Library size (read counts) of negative controls and samples using package decontam in R. **B.** Prevalence (presence/absence) across samples of a sequence feature (OTU) in true positive samples is compared to the prevalence in negative controls to identify contaminants using package decontam. **C.** Rarefaction curves showing sufficient sequencing coverage for all samples in Chesapeake and Erie. Curves plateau indicating that more sequence reads do not contribute to adding new members to the community. All samples were rarefied to an even read depth of 2,000 reads based on these results.

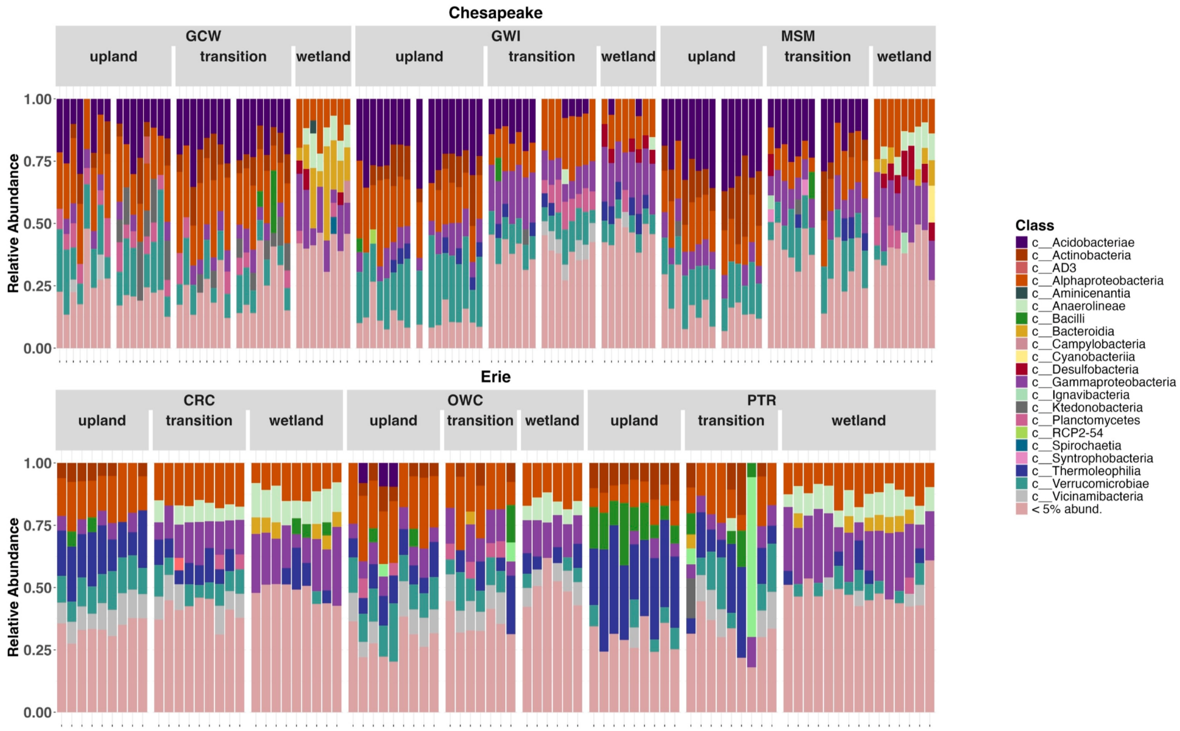

Fig. S4: Barplot showing bacterial community composition across all samples (at Class level) in Chesapeake and Erie.

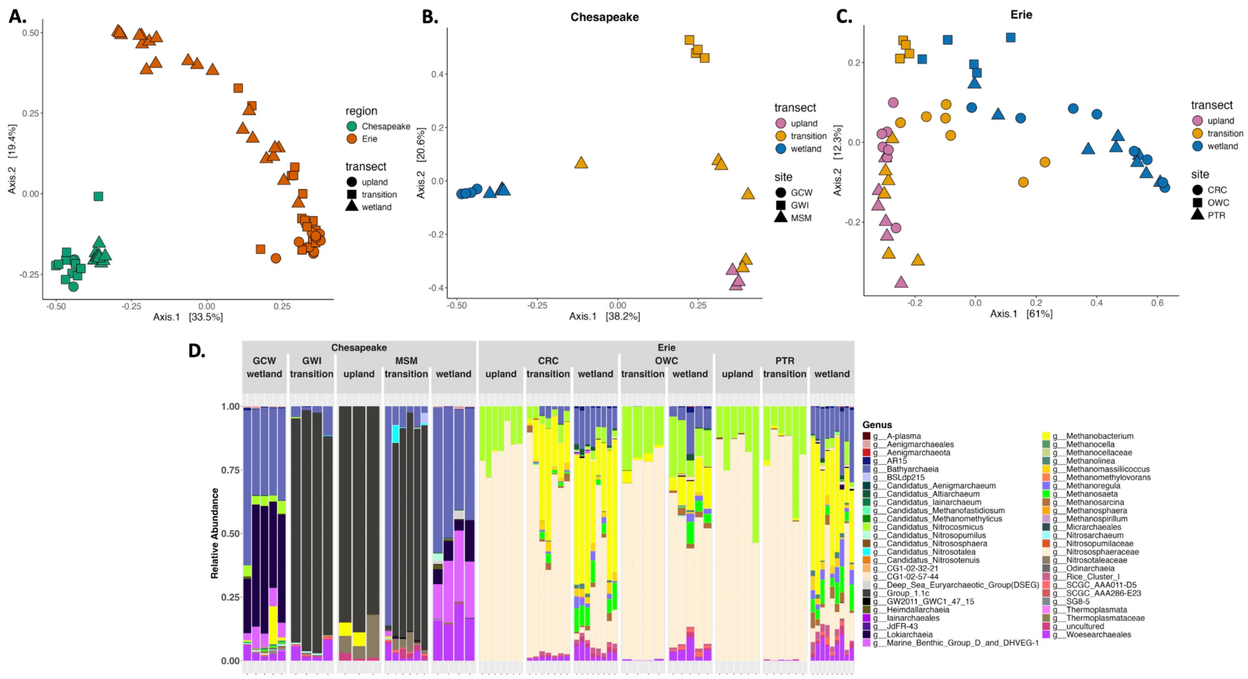

Fig. S5: Beta diversity of archaeal communities in Chesapeake and Erie by **A.** region, **B.** transect and site in Chesapeake, **C.** transect and site in Erie. D. Barplot showing archaeal community composition across all samples at genus level.

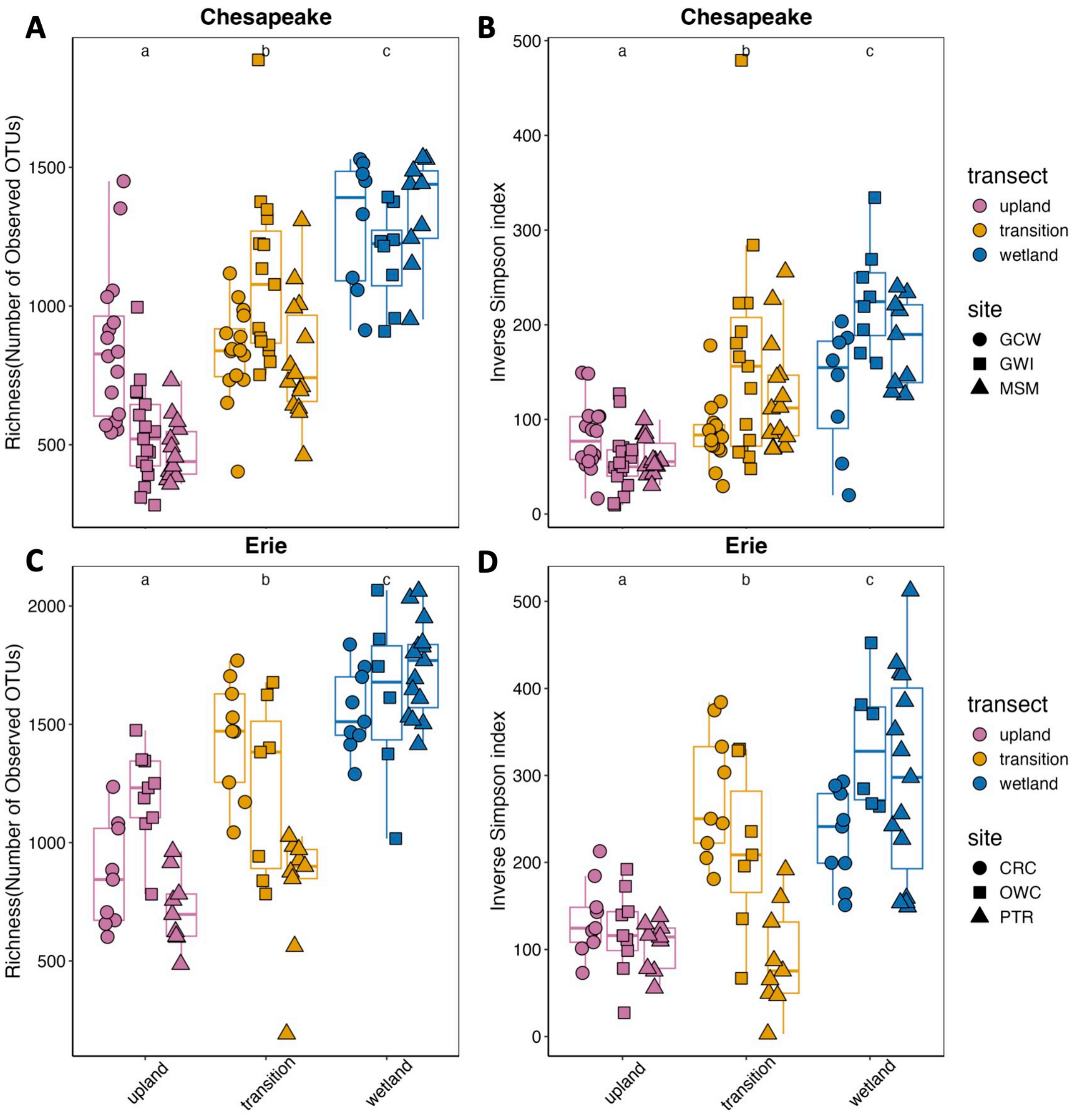

Fig. S6: Alpha diversity metrics (**A, C**) Richness (number of observed OTUs) and (**B, D**) diversity (Inverse Simpson Index) across transects and sites in Chesapeake and Erie. Transect positions with different letters represent statistically different values (P≤0.05).

A. Chesapeake

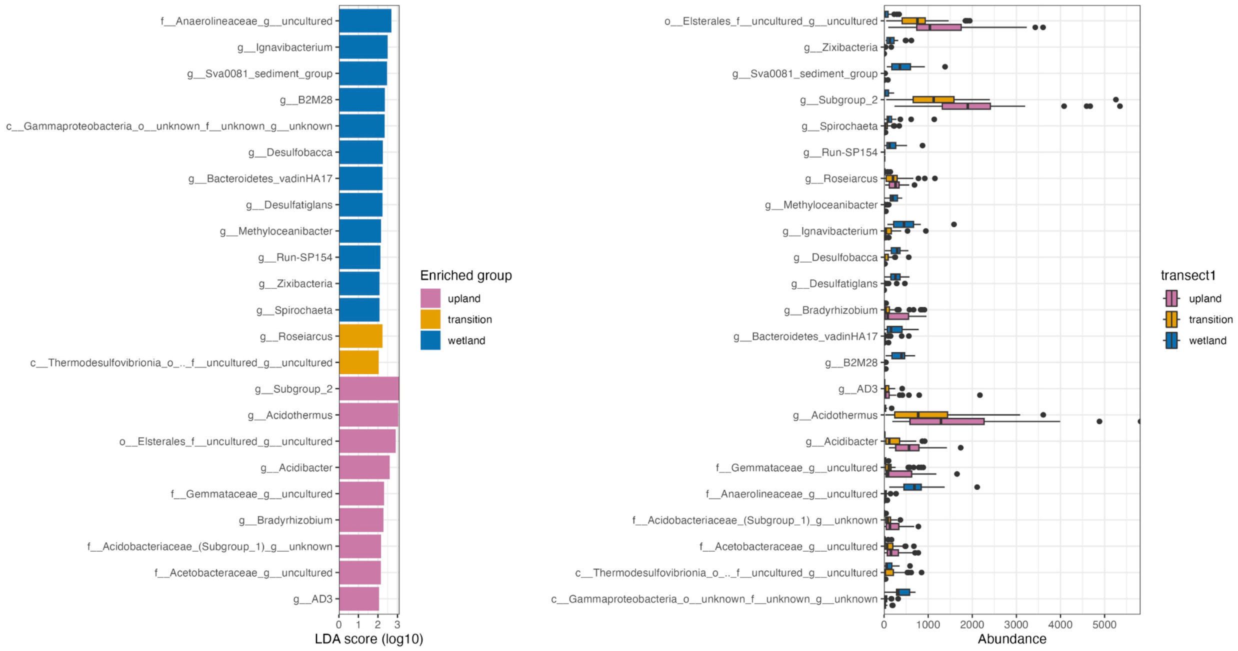

B. Erie

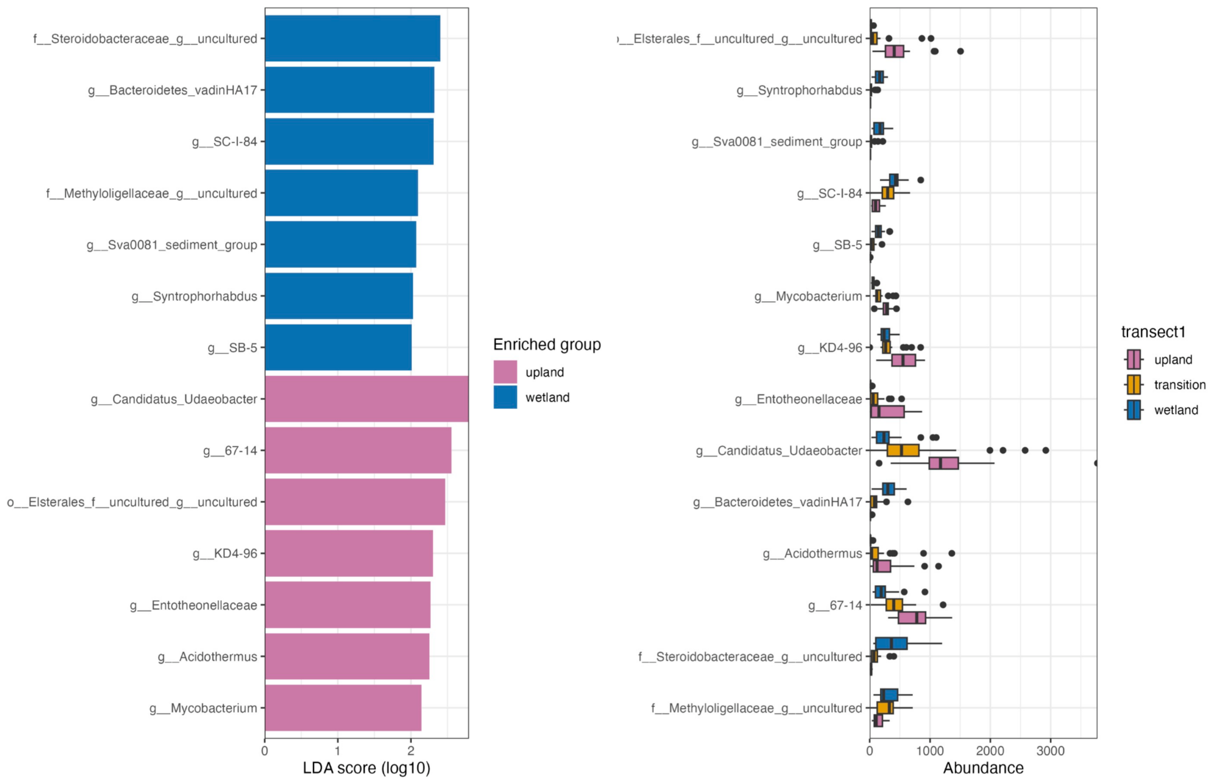

Fig. S7. LDA effect size (LEfSe) analysis determining bacterial genera most likely to explain differences between transects in **A.** Chesapeake, **B.** Erie. The LDA scores (left panel) are sorted by decreasing effect size.

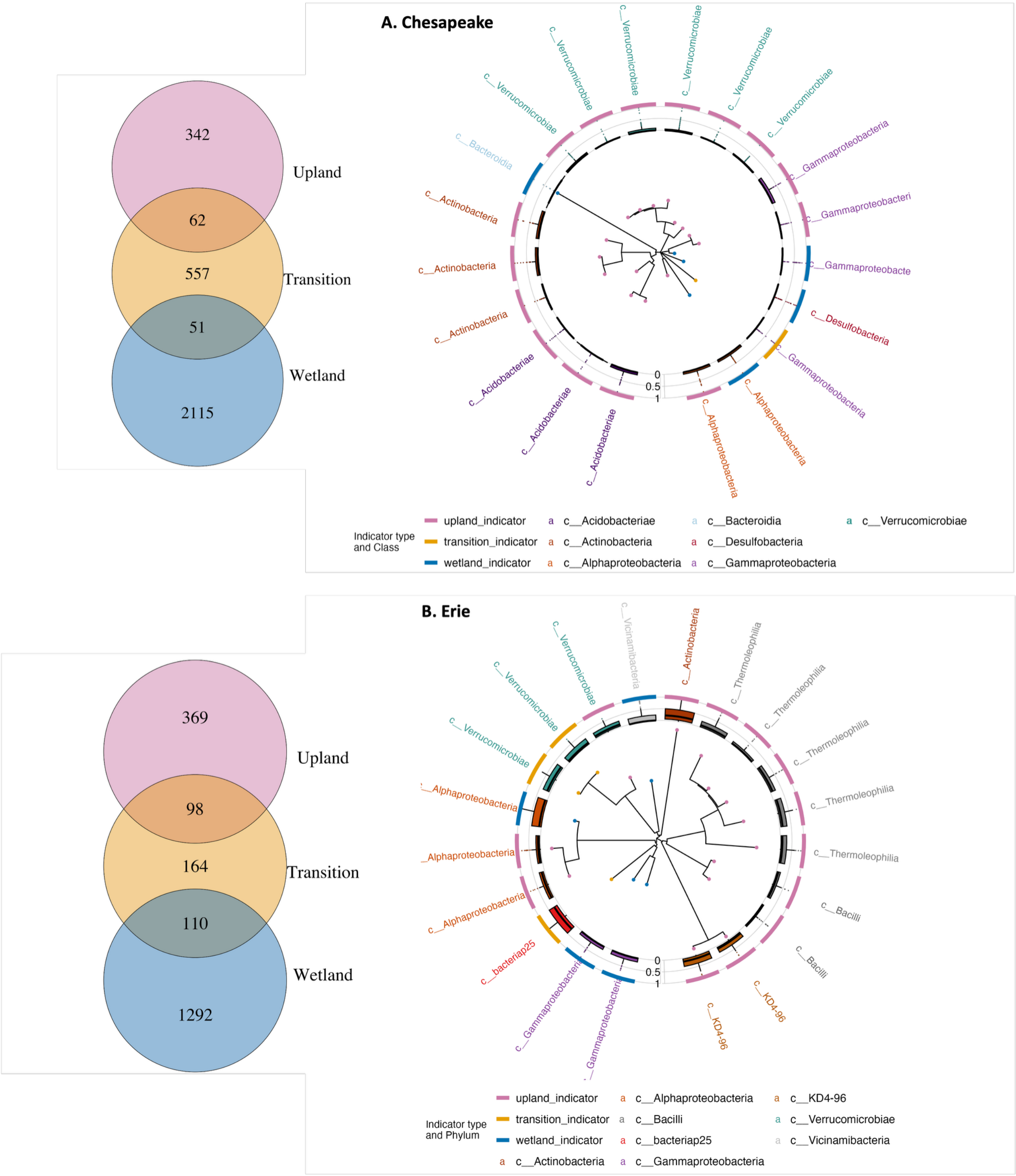

Fig. S8: Shared and unique indicator OTUs across transects and phylogenetic trees depicting 20 most abundant and unique indicator OTUs for A. Chesapeake and B. Erie. Family-level

designations of indicator OTUs are provided in the tree with genus-level classification in Table 5 of Supplementary Data. Phylum level classification is denoted in the legend.

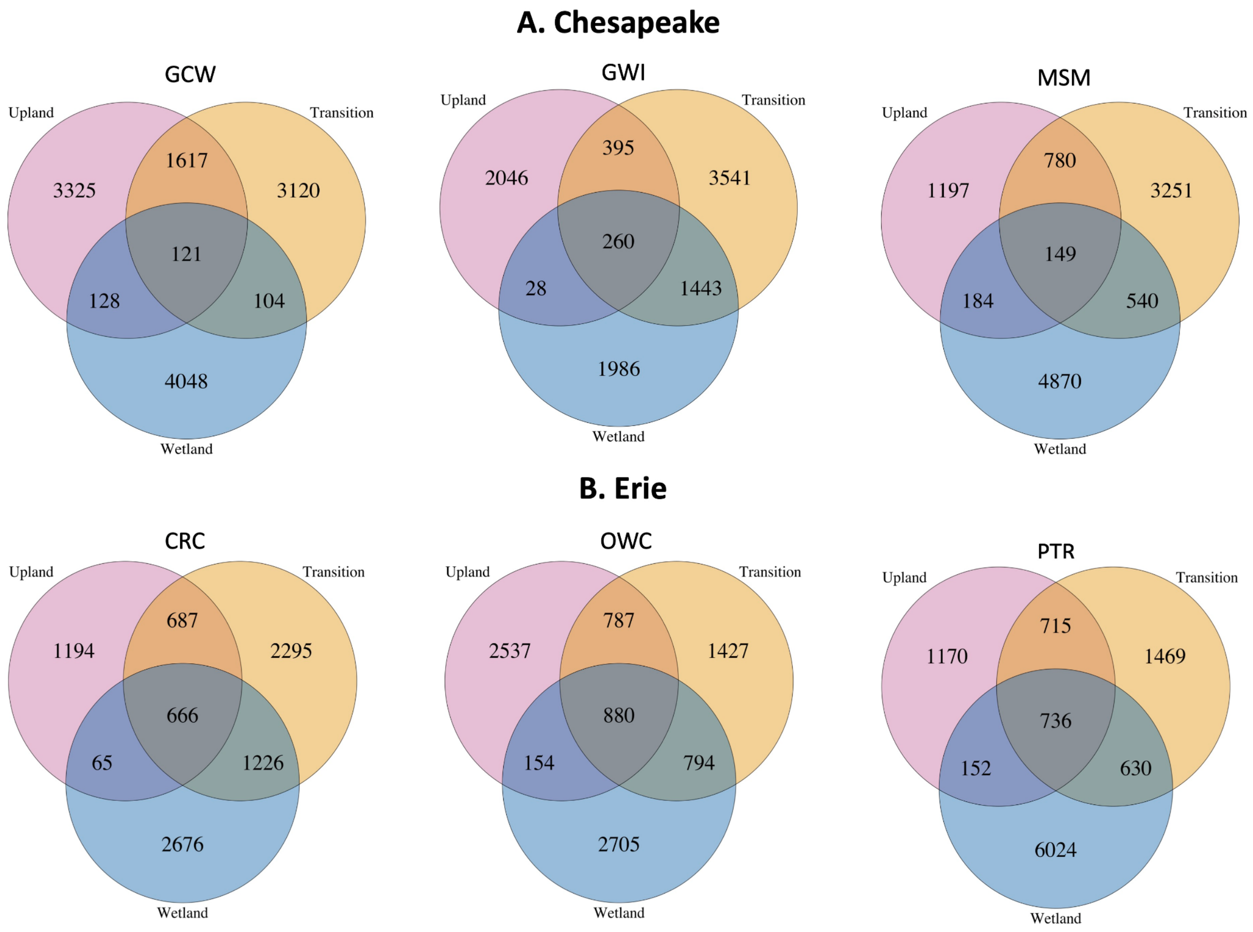

Fig. S9: Total number of shared and unique OTUs across transects within **A.** Chesapeake and **B.** Erie sites.

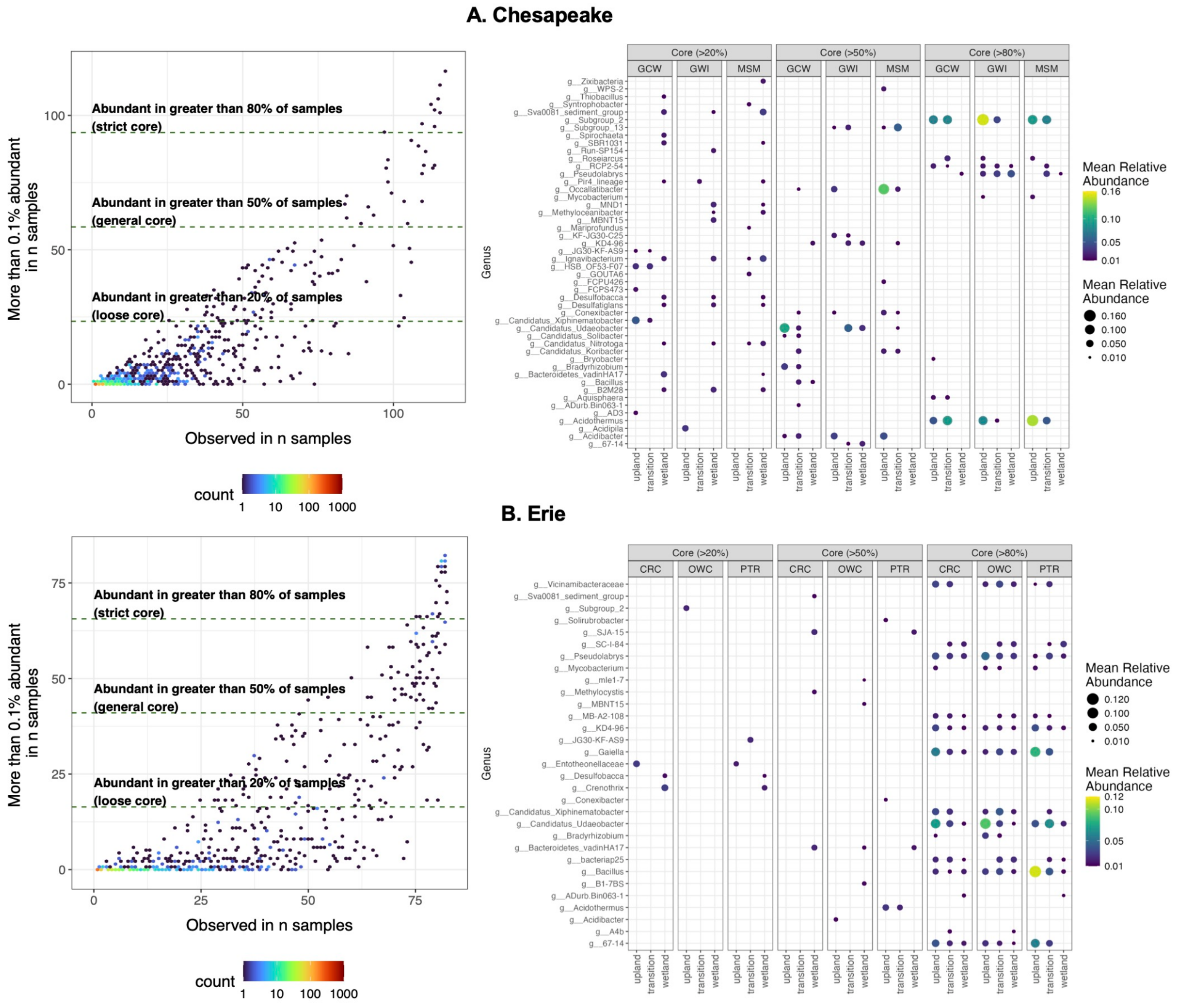

Fig. S10: Distribution of core microbiome members (left panels) and their relative abundances (right panels) across transects and sites in A. Chesapeake and B. Erie. Color gradients in the scatter plots (left panels) indicate the number of genera that satisfy the condition of being observed in n samples and having more than 0.1% abundance in n samples. Dot plots (right panels) show the mean relative abundance of core members along the TAI (abundance filtered to greater than 1% across samples). R script to assess core microbiome members was adapted from Dueholm et al. 2022.

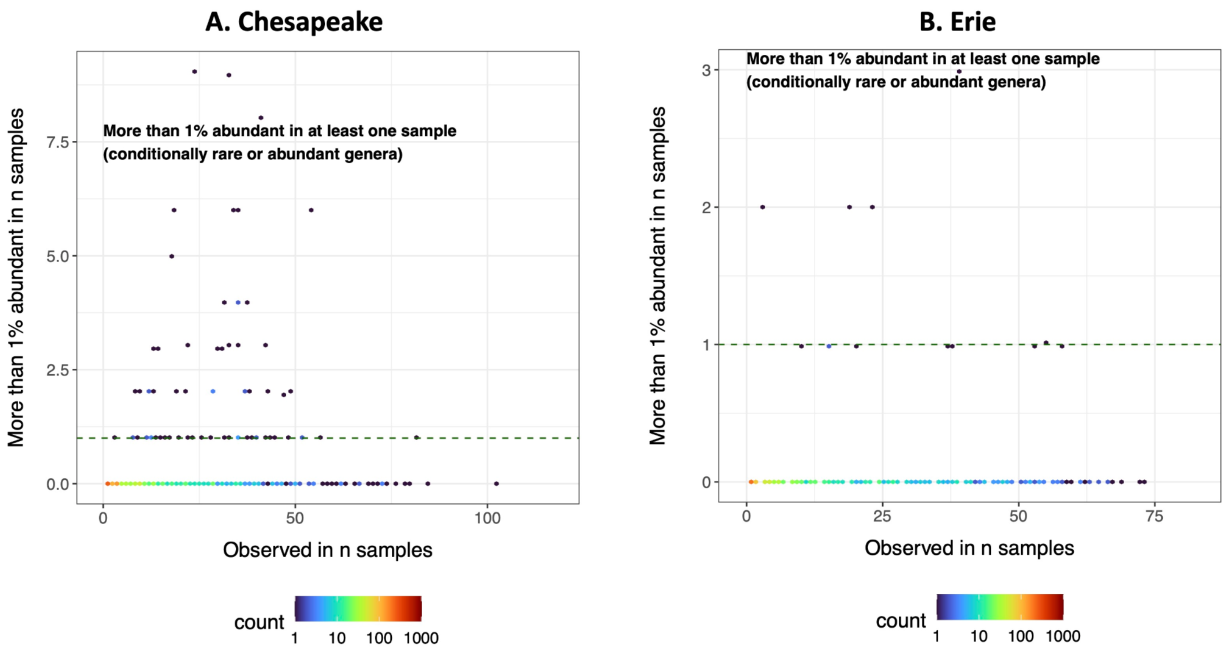

Fig. S11: Distribution of conditionally rare and abundant genera in **A.** Chesapeake and **B.** Erie.

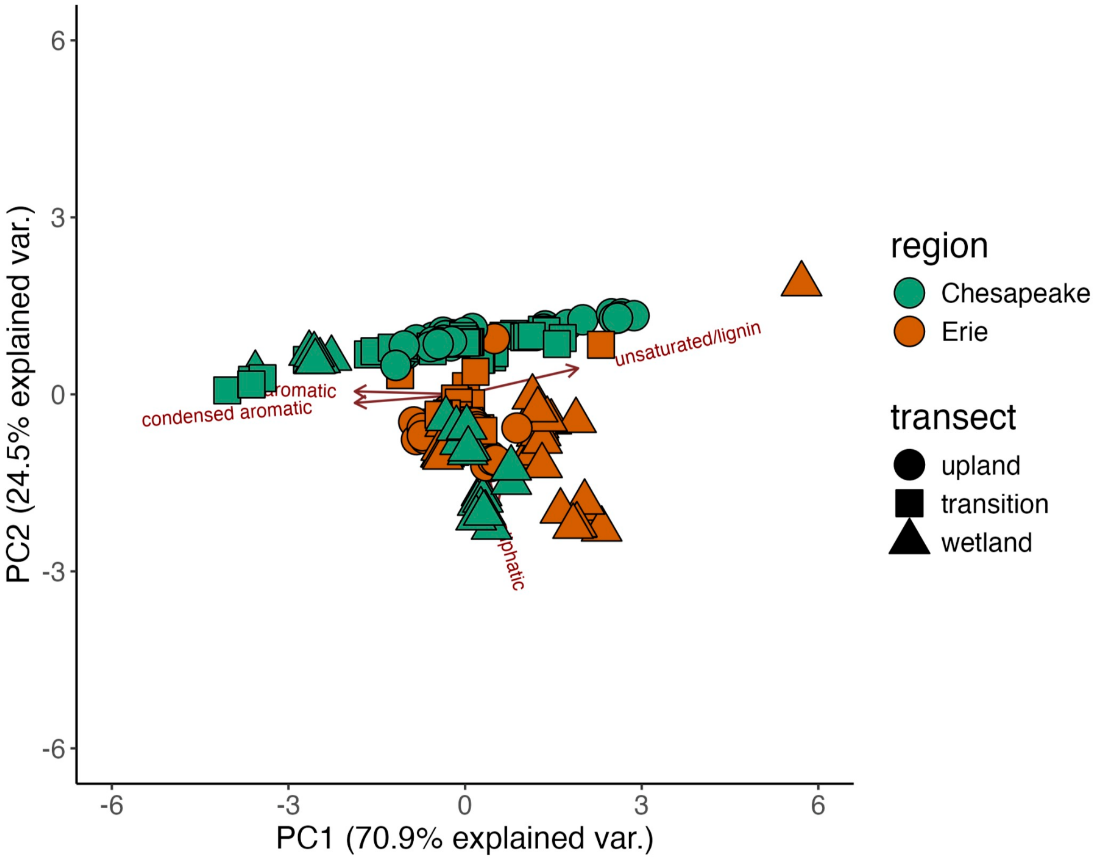

Fig. S12: Soil organic matter characterization using FTICR-MS for Chesapeake and Erie. PCA biplots depicting the organic compound classes that strongly influence the samples in Chesapeake and Erie.

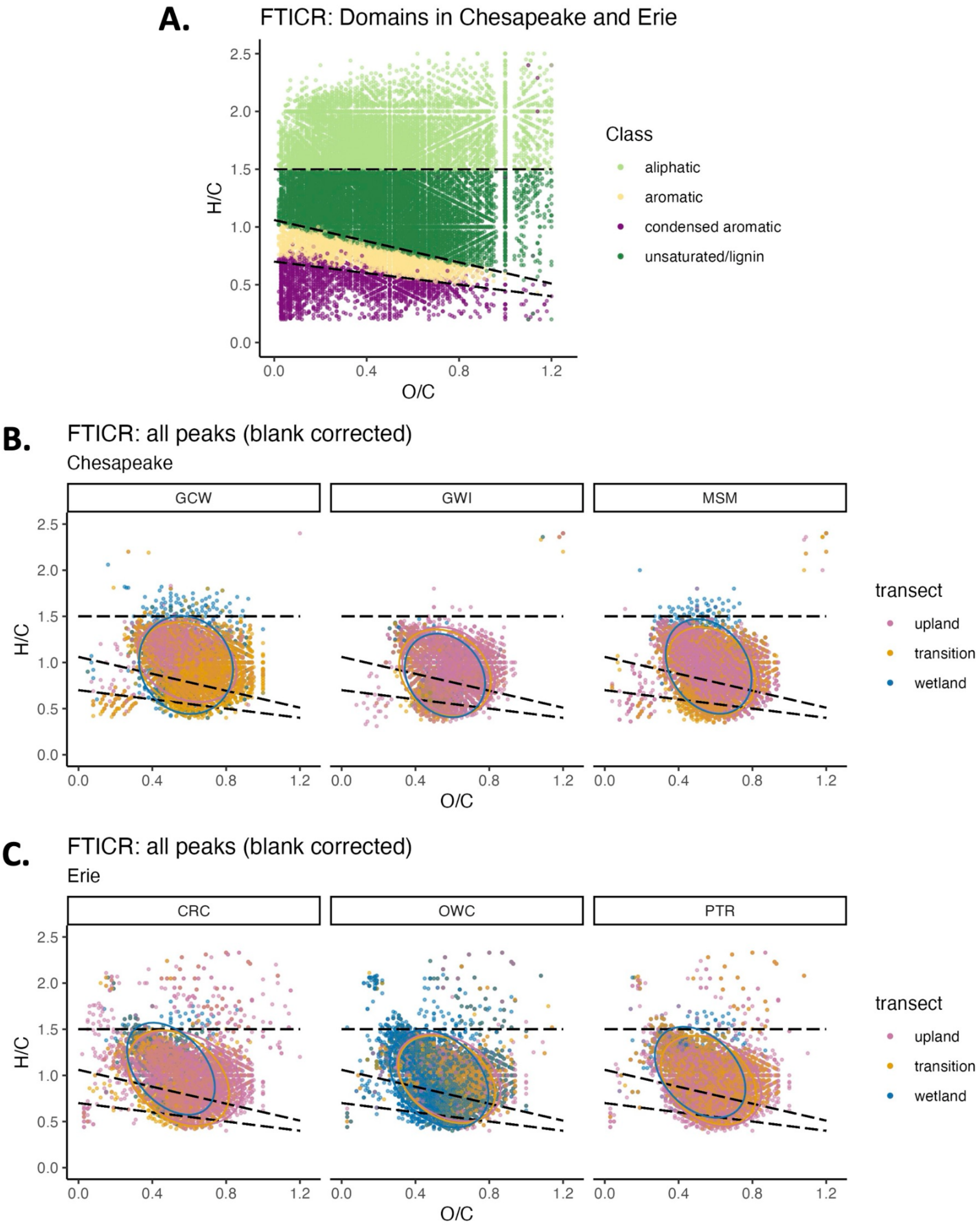

Fig. S13: A. Designation of organic compound classes in Chesapeake and Erie along a Van Krevelen plot. **B, C.** Van Krevlen plots depicting the distribution of the total chemical features

(within classes of aliphatic, aromatic, condensed aromatic, and unsaturated or lignin-like molecules) across transects and sites in **B.** Chesapeake and **C.** Erie. Presence-absence data are used for individual features instead of true abundance.

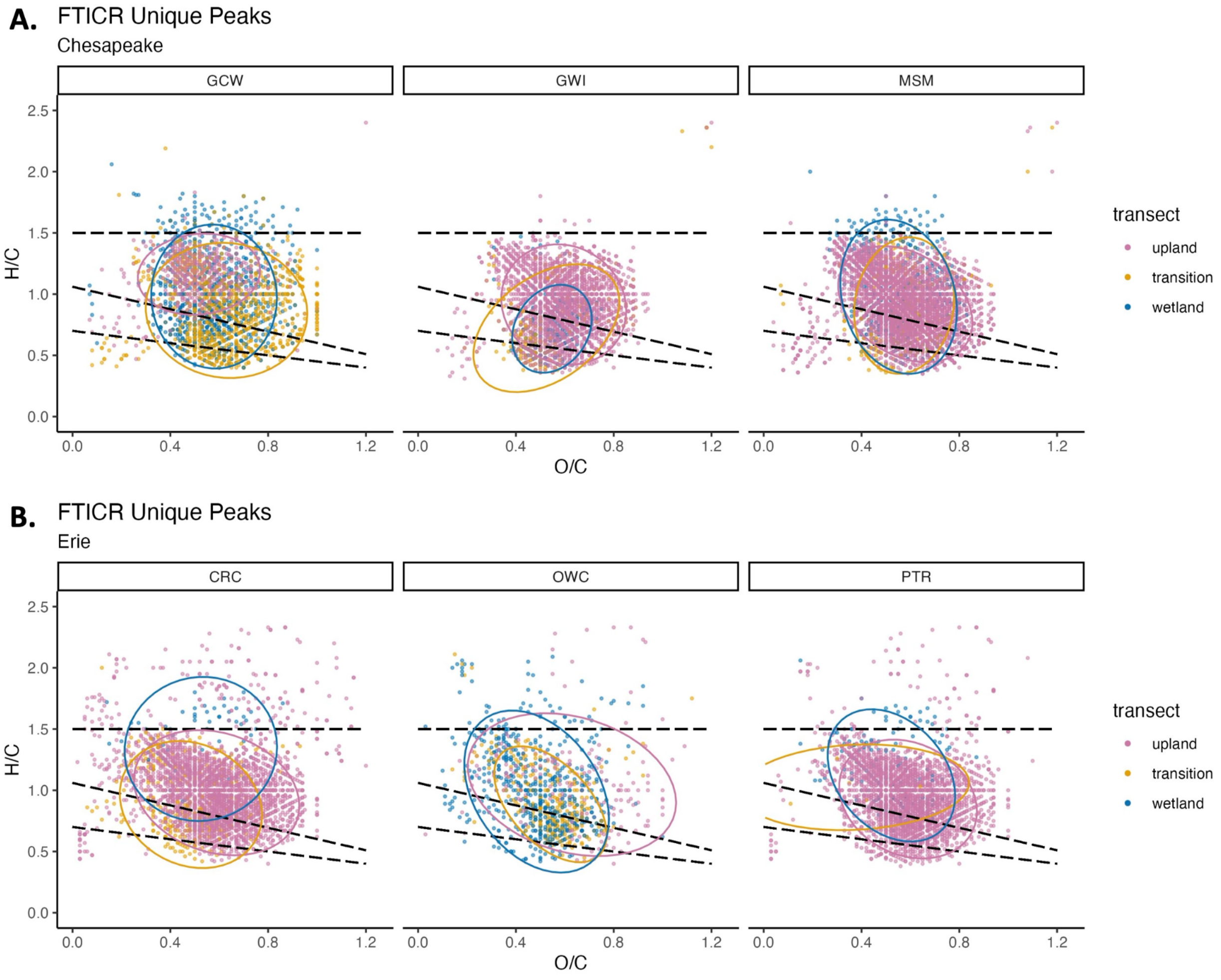

Fig. S14: Van Krevelen plots depicting the distribution of the unique chemical features (within classes of aliphatic, aromatic, condensed aromatic, and unsaturated or lignin-like molecules) across transects and sites in **A.** Chesapeake and **B.** Erie.

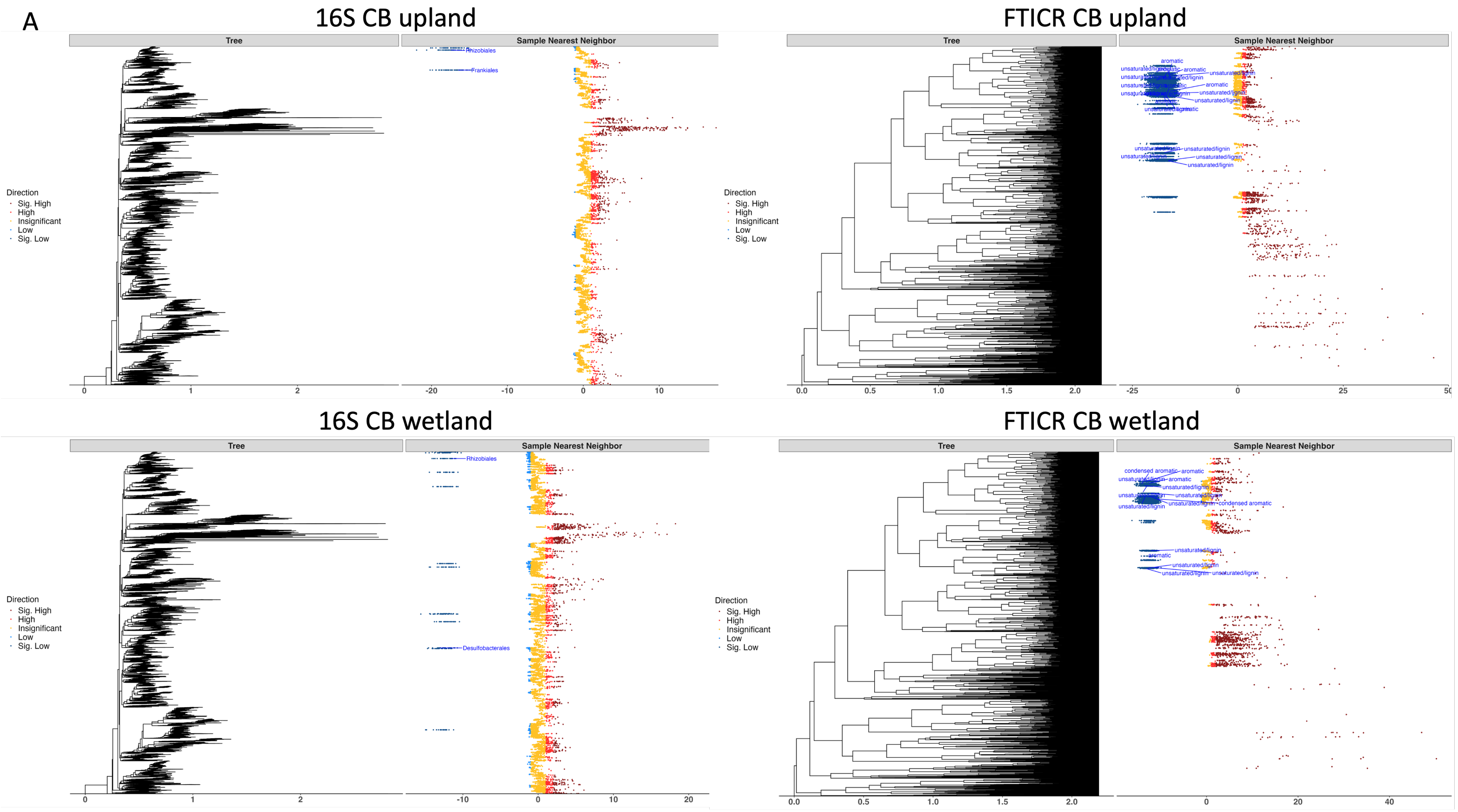
A
16S CB upland
FTICR CB upland
16S CB wetland
FTICR CB wetland
Tree
Sample Nearest Neighbor
Direction
Sig. High
High
Insignificant
Low
Sig. Low
Rhizobiales
Frankiales
Desulfobacterales
aromatic
unsaturated/lignin
condensed aromatic

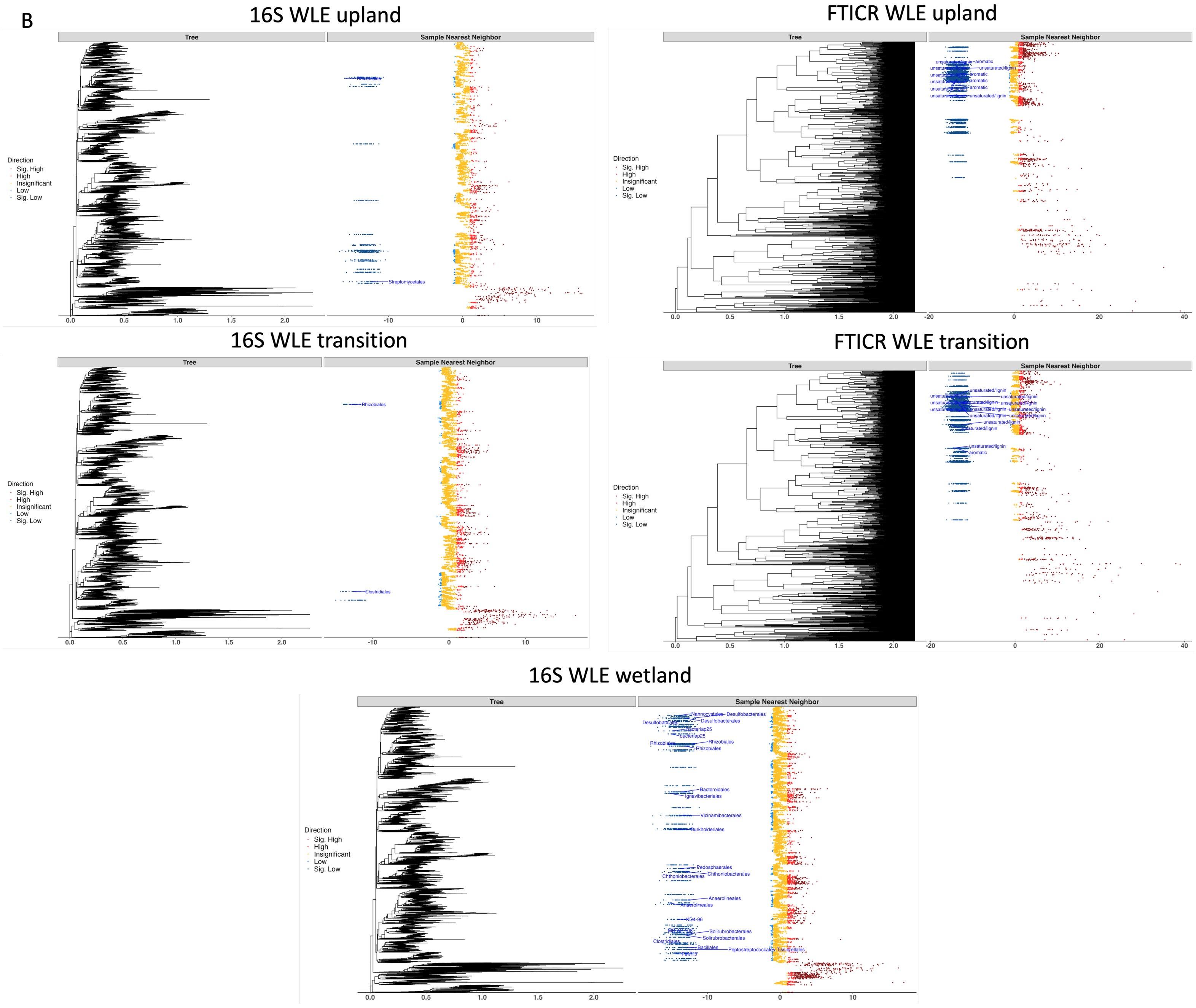
B
16S WLE upland
FTICR WLE upland
16S WLE transition
FTICR WLE transition
16S WLE wetland
Tree
Sample Nearest Neighbor
Direction
Sig. High
High
Insignificant
Low
Sig. Low
Streptomycetales
Rhizobiales
Clostridiales
unsaturated/lignin
aromatic
Nannocystales
Desulfobacterales
Bacteroidales
Ignavibacteriales
Vicinamibacterales
Burkholderiales
Pedosphaerales
Chthoniobacterales
Anaerolineales
KD4-96
Solirubrobacterales
Bacillales
Peptostreptococcales-Tissierellales
0.0
0.5
1.0
1.5
2.0
-10
0
10
-20
20
40

Fig. S15: Distribution of feature level βNTI_{feat} values for the 16S amplicon and FTICR data in **A.** Chesapeake and **B.** Erie transect positions. The values of βNTI_{feat} indicate whether a feature has an insignificant contribution to ecological variation across the metacommunity ($|\beta NTI_{feat}| < 1$), somewhat contributes to ecological variation ($1 < |\beta NTI_{feat}| < 2$), or significantly contributes to ecological variation ($|\beta NTI_{feat}| > 2$). When βNTI_{feat} trends are negative (i.e., < -1), the feature is expected to contribute to convergence across the data. When βNTI_{feat} trends are positive (i.e., >1), the feature is expected to contribute to divergence.

References

Chytrý, M., Tichý, L., Holt, J., Botta-Dukát, Z., 2002. Determination of diagnostic species with statistical fidelity measures. Journal of Vegetation Science: Official Organ of the International Association for Vegetation Science 13, 79–90.

Danczak, R.E., Chu, R.K., Fansler, S.J., Goldman, A.E., Graham, E.B., Tfaily, M.M., Toyoda, J., Stegen, J.C., 2020. Using metacommunity ecology to understand environmental metabolomes. Nature Communications 11, 6369.

Dittmar, T., Koch, B., Hertkorn, N., Kattner, G., 2008. A simple and efficient method for the solid-phase extraction of dissolved organic matter (SPE-DOM) from seawater: SPE-DOM from seawater. Limnology and Oceanography, Methods 6, 230–235.

Dueholm, M.K.D., Nierychlo, M., Andersen, K.S., Rudkjøbing, V., Knutsson, S., Arriaga, S., Bakke, R., Boon, N., Bux, F., Christensson, M., Chua, A.S.M., Curtis, T.P., Cytryn, E., Erijman, L., Etchebehere, C., Fatta-Kassinos, D., Frigon, D., Garcia-Chaves, M.C., Gu, A.Z., Horn, H., Jenkins, D., Kreuzinger, N., Kumari, S., Lanham, A., Law, Y., Leiknes, T., Morgenroth, E., Muszyński, A., Petrovski, S., Pijuan, M., Pillai, S.B., Reis, M.A.M., Rong, Q., Rossetti, S., Seviour, R., Tooker, N., Vainio, P., van Loosdrecht, M., Vikraman, R., Wanner, J., Weissbrodt, D., Wen, X., Zhang, T., Nielsen, Per H., Albertsen, M., Nielsen, Per Halkjær, Mi, D. A. S. Global Consortium, 2022. MiDAS 4: A global catalogue of full-length 16S rRNA gene sequences and taxonomy for studies of bacterial communities in wastewater treatment plants. Nature Communications 13, 1908.

Kew, W., Myers-Pigg, A., Chang, C.H., Colby, S.M., Eder, J., Tfaily, M.M., Hawkes, J., Chu, R.K., Stegen, J.C., 2024. Reviews and syntheses: Opportunities for robust use of peak intensities from high-resolution mass spectrometry in organic matter studies. Biogeosciences 21, 4665–4679.

Ko, D.K., Brandizzi, F., 2023. Coexpression Network Construction and Visualization from Transcriptomes Underlying ER Stress Responses. Methods in Molecular Biology (Clifton, N.J.) 2581, 385–401.

Koch, B.P., Dittmar, T., 2016. From mass to structure: an aromaticity index for high-resolution mass data of natural organic matter. Rapid Communications in Mass Spectrometry: RCM 30, 250–250.

Kujawinski, E., 2002. Electrospray ionization Fourier transform ion cyclotron resonance mass spectrometry (ESI FT-ICR MS): Characterization of complex environmental mixtures. Environmental Forensics 3, 207–216.

Kujawinski, E.B., Behn, M.D., 2006. Automated analysis of electrospray ionization fourier transform ion cyclotron resonance mass spectra of natural organic matter. Analytical Chemistry 78, 4363–4373.

Ohno, T., Sleighter, R.L., Hatcher, P.G., 2016. Comparative study of organic matter chemical characterization using negative and positive mode electrospray ionization ultrahigh-resolution mass spectrometry. Analytical and Bioanalytical Chemistry 408, 2497–2504.

Patel, K.F., 2020. kaizadp/fticrrr: FTICR-results-in-R. Zenodo. doi:10.5281/ZENODO.3893246

Patel, K.F., Malhotra, A., Norris, C.G., McKever, S.A., Fields, D.M., Musci, J.I., Bandopadhyay, S., Bond-Lamberty, B., Chen, X., Day, D.J., Doro, K.O., Fluet-Chouinard, E., Garcia, M., Kemner, K.M., Machado-Silva, F., McDowell, N., Morris, K.A., Myers-Pigg, A., O'Loughlin, E.J., O'Meara, T., Peixoto, R.B., Pennington, S.C., Regier, P., Rich, R., Rod, K.A., Sulman, B., Thornton, P., Ward, N., Wilson, S.J., Weintraub, M.N.,

Megonigal, J.P., Bailey, V.L., 2025. Transition zones at the changing coastal terrestrial-aquatic interface. Journal of Geophysical Research. Biogeosciences 130, e2025JG008978.
Patel, K.F., Myers-Pigg, A., Bond-Lamberty, B., Fansler, S.J., Norris, C.G., McKever, S.A., Zheng, J., Rod, K.A., Bailey, V.L., 2021. Soil carbon dynamics during drying vs. rewetting: Importance of antecedent moisture conditions. Soil Biology & Biochemistry 156, 108165.
Payne, T.G., Southam, A.D., Arvanitis, T.N., Viant, M.R., 2009. A signal filtering method for improved quantification and noise discrimination in fourier transform ion cyclotron resonance mass spectrometry-based metabolomics data. Journal of the American Society for Mass Spectrometry 20, 1087–1095.
Seidel, M., Beck, M., Riedel, T., Waska, H., Suryaputra, I.G.N.A., Schnetger, B., Niggemann, J., Simon, M., Dittmar, T., 2014. Biogeochemistry of dissolved organic matter in an anoxic intertidal creek bank. Geochimica et Cosmochimica Acta 140, 418–434.
Seidel, M., Manecki, M., Herlemann, D.P.R., Deutsch, B., Schulz-Bull, D., Jürgens, K., Dittmar, T., 2017. Composition and transformation of dissolved organic matter in the Baltic sea. Frontiers in Earth Science 5, 241419.
Sleighter, R.L., Chen, H., Wozniak, A.S., Willoughby, A.S., Caricasole, P., Hatcher, P.G., 2012. Establishing a measure of reproducibility of ultrahigh-resolution mass spectra for complex mixtures of natural organic matter. Analytical Chemistry 84, 9184–9191.
Tfaily, M.M., Chu, R.K., Tolić, N., Roscioli, K.M., Anderton, C.R., Paša-Tolić, L., Robinson, E.W., Hess, N.J., 2015. Advanced solvent based methods for molecular characterization of soil organic matter by high-resolution mass spectrometry. Analytical Chemistry 87, 5206–5215.
Tfaily, M.M., Chu, R.K., Toyoda, J., Tolić, N., Robinson, E.W., Paša-Tolić, L., Hess, N.J., 2017. Sequential extraction protocol for organic matter from soils and sediments using high resolution mass spectrometry. Analytica Chimica Acta 972, 54–61.
Tolić, N., Liu, Y., Liyu, A., Shen, Y., Tfaily, M.M., Kujawinski, E.B., Longnecker, K., Kuo, L.-J., Robinson, E.W., Paša-Tolić, L., Hess, N.J., 2017. Formularity: Software for automated formula assignment of natural and other organic matter from ultrahigh-resolution mass spectra. Analytical Chemistry 89, 12659–12665.
Zhang, B., Horvath, S., 2005. A general framework for weighted gene co-expression network analysis. Statistical Applications in Genetics and Molecular Biology 4.
